# Transcription of the three HMG-CoA reductase genes of *Mucor circinelloides*

**DOI:** 10.1186/1471-2180-14-93

**Published:** 2014-04-14

**Authors:** Gábor Nagy, Anita Farkas, Árpád Csernetics, Ottó Bencsik, András Szekeres, Ildikó Nyilasi, Csaba Vágvölgyi, Tamás Papp

**Affiliations:** 1 Department of Microbiology, Faculty of Science and Informatics, University of Szeged, Közép fasor 52, Szeged, H-6726, Hungary

**Keywords:** Terpene biosynthesis, Transcription, Ergosterol, Carotenoid, Dimorphism, Mucorales

## Abstract

**Background:**

Precursors of sterols, carotenoids, the prenyl groups of several proteins and other terpenoid compounds are synthesised via the acetate-mevalonate pathway. One of the key enzyme of this pathway is the 3-hydroxy-3-methylglutaryl-CoA (HMG-CoA) reductase, which catalyses the conversion of HMG-CoA to mevalonate. HMG-CoA reductase therefore affects many biological processes, such as morphogenesis, synthesis of different metabolites or adaptation to environmental changes. In this study, transcription of the three HMG-CoA reductase genes (designated as *hmgR1*, *hmgR2* and *hmgR3*) of the β-carotene producing *Mucor circinelloides* has been analysed under various culturing conditions; effect of the elevation of their copy number on the carotenoid and ergosterol content as well as on the sensitivity to statins has also been examined.

**Results:**

Transcripts of each gene were detected and their relative levels varied under the tested conditions. Transcripts of *hmgR1* were detected only in the mycelium and its relative transcript level seems to be strongly controlled by the temperature and the oxygen level of the environment. Transcripts of *hmgR2* and *hmgR3* are already present in the germinating spores and the latter is also strongly regulated by oxygen. Overexpression of *hmgR2* and *hmgR3* by elevating their copy numbers increased the carotenoid content of the fungus and decreased their sensitivity to statins.

**Conclusions:**

The three HMG-CoA reductase genes of *M. circinelloides* displayed different relative transcript levels under the tested conditions suggesting differences in their regulation. They seem to be especially involved in the adaptation to the changing oxygen tension and osmotic conditions of the environment as well as to statin treatment. Overexpression of *hmgR2* and *hmgR3* may be used to improve the carotenoid content.

## Background

*Mucor circinelloides* (Mucoromycotina, Mucorales) is a β-carotene producing fungus and, besides the related *Blakeslea trispora* and *Phycomyces blakesleeanus*, it has been used as a model organism in carotenogenic studies [1-3] Carotenoids and other terpenoids of the fungal cell, such as ergosterol or the prenyl groups of ubiquinone and the farnesylated and geranylgeranylated proteins, are synthesised via the acetate-mevalonate pathway, in which the conversion of 3-hydroxy-3-methylglutaryl-CoA (HMG-CoA) to mevalonate is thought to be a rate-limiting step [4,5]. This reaction is catalysed by the HMG-CoA reductase (EC 1.1.1.88), which is therefore considered to be a key enzyme of the terpenoid biosynthesis. Characterization of this enzyme and the encoding gene can serve valuable information for the exploitation and improvement of the carotenoid production by Mucoral fungi. At the same time, these studies may lead us to better understand various physiological and cell biological processes, such as morphogenesis, synthesis of different metabolites, response to environmental changes (e.g. changes in the oxygen concentration or salinity of the environment) or fungal pathogenesis, in which HMG-CoA has been found to have a central role [5-9] Via its participation in the terpenoid biosynthesis, HMG-CoA reductase affects so important features of the fungal cell as ergosterol content, membrane structure and fluidity or signalling and regulation processes (e.g. via the prenylation of proteins). Moreover, ergosterol is an important component of the fungal cell membrane and the main target of the clinically used antifungal agents. Previously, it was reported that treatment with lovastatin, a competitive inhibitor of HMG-CoA reductase, caused altered processing of MRas1 protein, blockage of MRas3 accumulation, decreased growth and sporangiospore germination and apoptosis-like cell death in *Mucor*[6].

In the *M. circinelloides* genome, three genes that potentially encode HMG-CoA reductases can be identified [10]. However, information on their role in the different biological processes has not been available until to date. Therefore, the aim of the present study was to detect differences in their transcript levels and responses to changes of various environmental conditions, which may be relevant from both physiological and biotechnological points of view, such as growth temperature, salinity of the medium, carbon source and oxygen tension. Another objective was to examine whether any of the three genes have a special effect on the carotenoid and ergosterol content of the fungus. To answer these questions, transcription of the genes was analysed by real-time quantitative PCR (qPCR) and mutant strains overexpressing the different isogenes were also constructed.

## Results

### HMG-CoA reductases of *M. circinelloides*

Using Blast searches in the *Mucor* genome database (DoE Joint Genome Institute; *M. circinelloides* CBS277.49v2.0; http://genome.jgi-psf.org/Mucci2/Mucci2.home.html), three potential HMG-CoA reductase genes were found at the following locations, scaffold_02: 2759562-2763160; scaffold_03: 4299175-4302130 and scaffold_04: 4237143-4240758 and named as *hmgR1*, *hmgR2* and *hmgR3*, respectively. Based on these sequence data, specific primers were designed (Additional file 1: Table S1) and the three genes and the appropriate cDNAs were isolated. The gene sequences were deposited to NCBI GenBank (accession numbers for *hmgR1*, *hmgR2* and *hmgR3* are KJ508882, KJ508884, and KJ508883, respectively.) The three *hmgR* genes encode the putative HmgR1, HmgR2 and HmgR3 proteins, which consist of 1107, 1078 and 1115 amino acids and have 120.78, 118.45 and 120.71 kDa, calculated molecular mass, respectively. Main features of the three proteins are summarized in Additional file 1: Table S2. HMG-CoA reductases are membrane-anchored proteins, wherein three main regions can be distinguished: the N-terminal hydrophobic domain containing several transmembrane segments [11], the conserved C-terminal catalytic domain and, between them, a short linker region. These regions, including the putative transmembrane helices and the sterol-sensing domain (SSD) in the N-terminal part, were identified (Additional file 1: Table S2). Number of the putative transmembrane domains varies in the three proteins, transmembrane domain prediction found six, nine and five transmembrane helices in HmgR1, HmgR2 and HmgR3, respectively. The putative pI of three proteins was found to be 8.86, 8.33 and 8.43 for HmgR1, HmgR2 and HmgR3, respectively. The HMG-CoA binding motif CENVIGYMPIP [12] and the two putative NAD(P)H binding sites were found in the catalytic domains of the three proteins (Additional file 1: Table S2). Ruiz-Albert et al. [13] predicted a short C-terminal PEST sequence as a signal for the rapid protein degradation in the HMG-CoA reductase of *P. blakesleeanus.* This signal was found in neither of the HMG-CoA reductases of *M. circinelloides*.

The whole HmgR1 protein shows 47 and 57% amino acid identity to HmgR2 and HmgR3, respectively, while HmgR2 and HmgR3 have a 55% overall identity to each other. At the same time, the catalytic domain of HmgR1 shows 80 and 81% sequence identity to those of HmgR2 and HmgR3, respectively; the catalytic domain of HmgR2 proved to be identical with that of HmgR3 in 87%. An alignment and comparison of the amino acid sequences of the three *Mucor* proteins are shown in (for an alignment with other HmgR sequences, see Additional file 1: Figure S1).

### Relative transcript levels of the *hmgR* genes during the cultivation period

**Figure 1:**
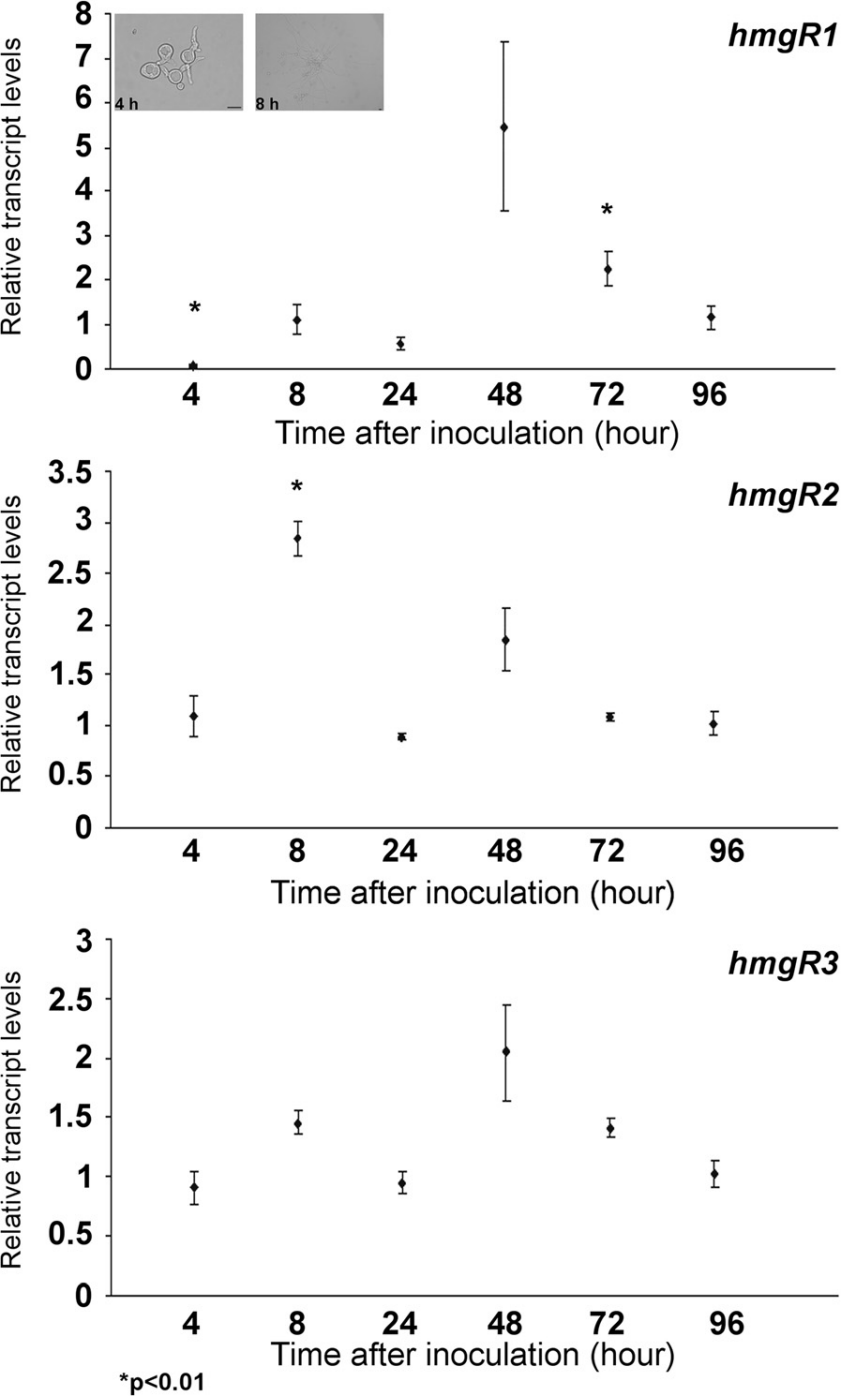
**Relative transcript levels of the *hmgR *genes of *M. circinelloides *during the cultivation period.** MS12 was grown on YNB under continuous light at 25°C; transcript level of each gene measured at 96 h was taken as 1. The presented values are averages of three independent experiments; error bars indicate standard deviation. Relative transcript values followed by * significantly differed from the value taken as 1 according to the paired *t*-test (p0.01). Light micrographs show the morphology of *Mucor circinelloides* germinating spores and young hyphae at 4 and 8 h postinoculation, respectively; scale bars indicate 10 μm.

RNA extractions were performed at different times of cultivation. At 4 h postinoculation, germ tubes are just developed while branched hyphae appear at about 8 h postinoculation (Figure 1). During the cultivation period, transcript levels of *hmgR2* and *hmgR3* showed similar patterns; both reached high amounts already at 4 h postinoculation indicating that these transcripts are present in the germinating spores (Figure 1). During the whole cultivation cycle, *hmgR2* showed the highest relative transcript level at 8 h postinoculation, while those of *hmgR1* and *hmgR3* reached their maximum values at 48 h after the inoculation. Additional file 1: Figure S2 shows the transcript levels of the genes relative to the transcript level of *hmgR1* at 96 hours. In comparison with the other genes, *hmgR2* showed the highest and *hmgR1* the lowest relative transcript levels during the whole cultivation cycle. Although *hmgR1* had very low transcript levels in all further experiments (Additional file 1: Figure S2 – S6), reverse transcription PCR clearly proved the transcription of all three genes (Additional file 1: Figure S7).

### Relative transcript levels of the *hmgR* genes at different temperatures

**Figure 2:**
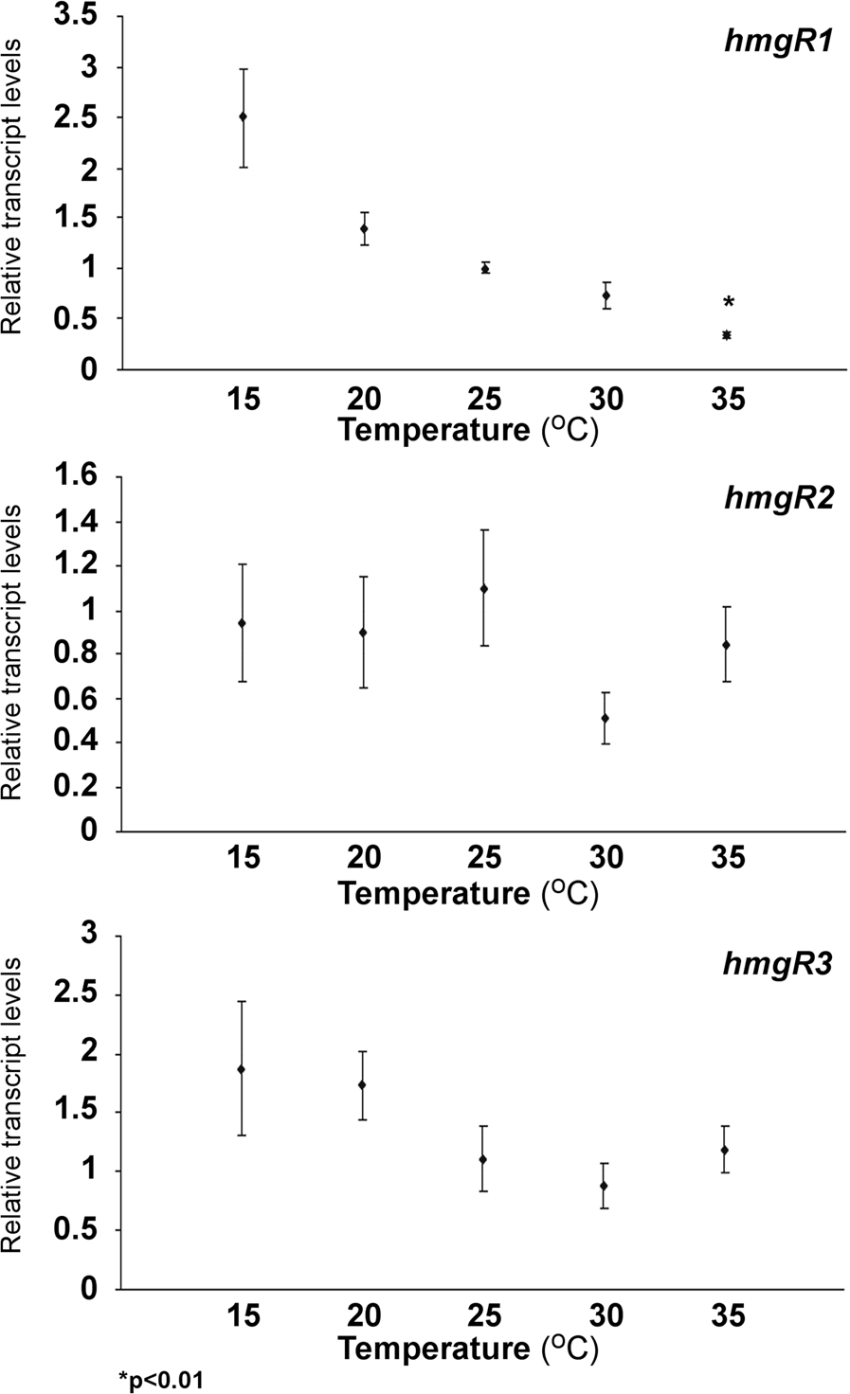
**Relative transcript levels of the *hmgR *genes of *M. circinelloides *at different cultivation temperatures.** MS12 was grown on YNB under continuous light for 4 days; transcript level of each gene measured at 25°C was taken as 1. The presented values are averages of three independent experiments; error bars indicate standard deviation. Relative transcript values followed by * significantly differed from the value taken as 1 according to the paired *t*-test (p < 0.01).

Relative transcript levels of the three *hmgR* genes detected after 4 days of cultivation at different temperatures are shown in Figure 2. The transcript abundance of *hmgR1* did not run parallel to that of the actin gene, but there was a steady relative decrease with the increasing growth temperatures. Apart from slight fluctuations, amounts of the transcripts of *hmgR2* and *hmgR3* did not changed at the different temperatures. Additional file 1: Figure S3 shows the transcript levels of the genes relative to the transcript level of *hmgR1* at 25°C.

### Effect of salt stress on the transcript levels of the *hmgR* genes

**Figure 3:**
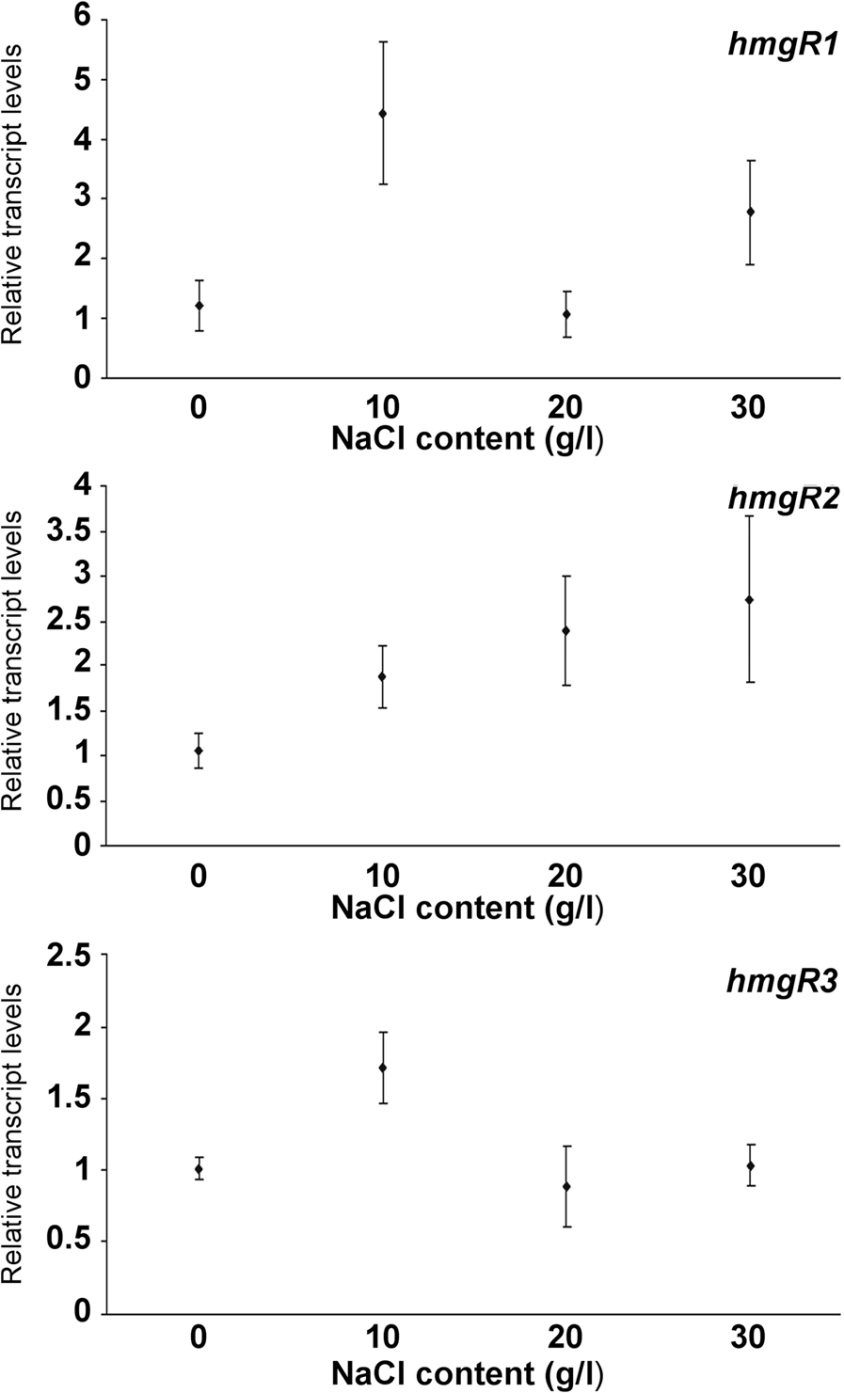
**Relative transcript levels of the *hmgR *genes of *M. circinelloides *at different salt concentrations.** MS12 was grown on YNB under continuous light for 4 days at 25°C; transcript level of each gene measured in the untreated control was taken as 1. The presented values are averages of three independent experiments; error bars indicate standard deviation.

*M. circinelloides* was grown for 4 days on YNB supplemented with NaCl to the final concentrations of 10, 20 and 30 g/l. Transcript levels of all three genes responded to the addition of NaCl into the culture medium. Transcript level of *hmgR1* increased in the presence of 10 g/l NaCl while that of *hmgR2* continuously increased with the elevating salt concentrations (Figure 3). Presentation of the transcript levels of the genes relative to that of the *hmgR1* in the untreated control indicates that *hmgR2* had extremely high relative transcript levels on salt containing media in comparison with the other two genes (Additional file 1: Figure S4).

### Relative transcript levels of the *hmgR* genes on different carbon sources

**Figure 4:**
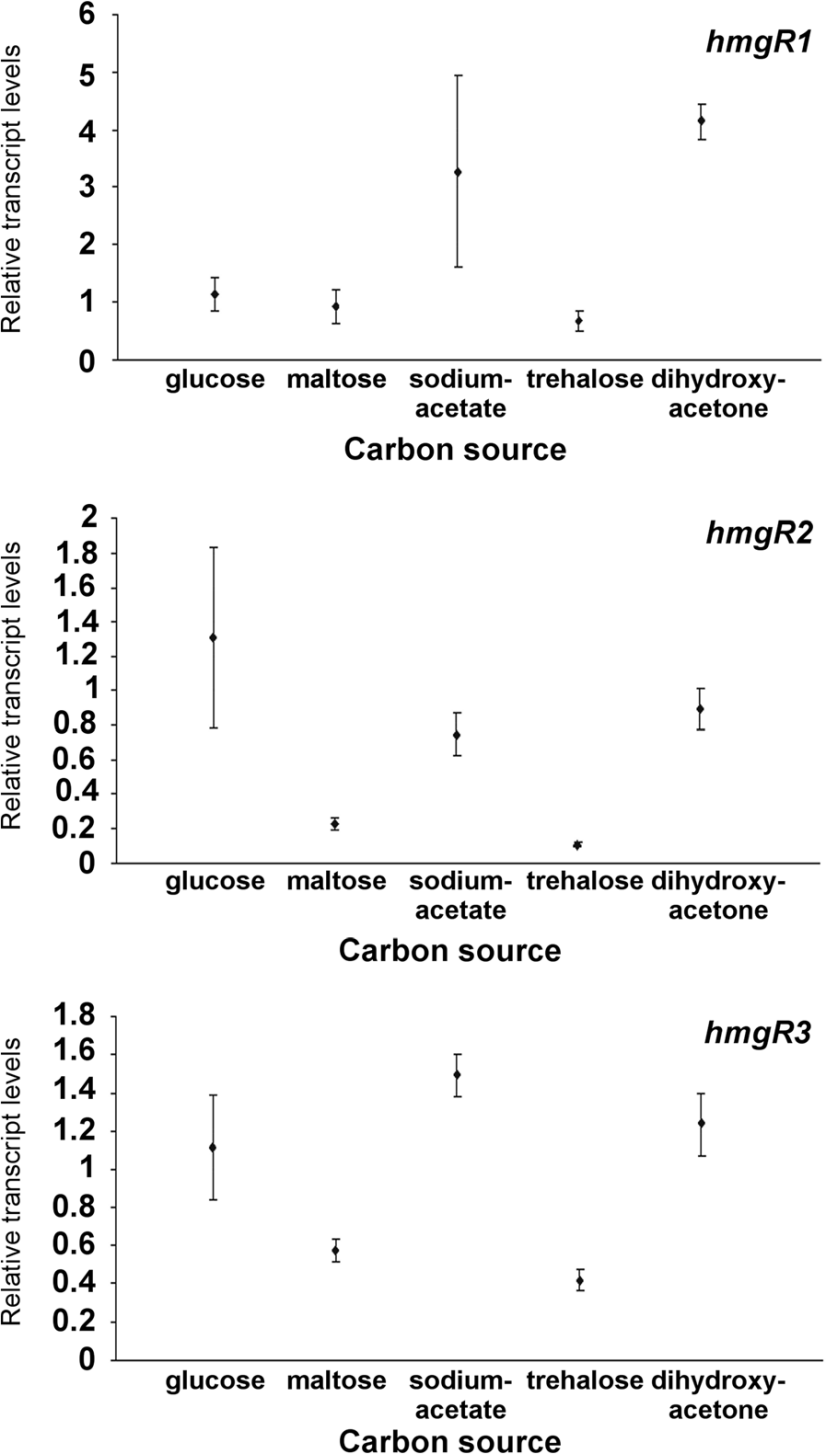
**Relative transcript levels of the *hmgR *genes of *M. circinelloides *growing on different carbon sources.** MS12 was grown on YNB containing different carbon sources under continuous light for 4 days at 25°C; transcript level of each gene measured on YNB with glucose was taken as 1. The presented values are averages of three independent experiments; error bars indicate standard deviation.

*M. circinelloides* was cultivated on solid YNB containing glucose, maltose, sodium acetate, trehalose, or dihydroxyacetone as single carbon sources for 4 days (Figure 4). All three genes displayed high transcript levels on sodium acetate and dihydroxyacetone. In case of *hmgR2* and *hmgR3*, glucose also induced high transcript abundance. Interestingly, relative transcript level of *hmgR1* on glucose did not differ significantly from those reached on maltose and trehalose. In comparison with the other genes, *hmgR2* displayed the highest relative transcript levels on all carbon sources (Additional file 1: Figure S5).

### Relative transcript levels of the *hmgR* genes under aerobic and anaerobic conditions

**Figure 5:**
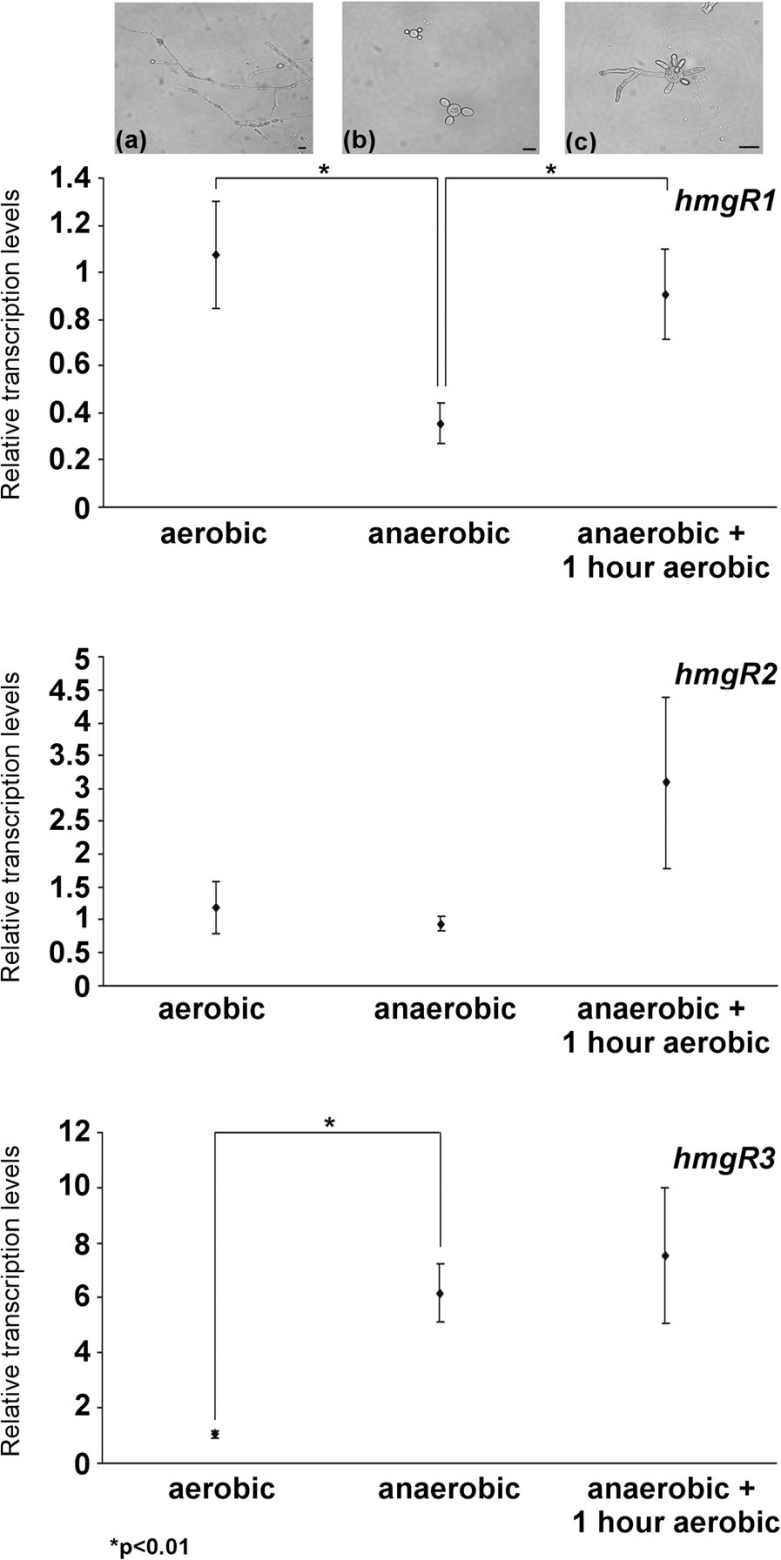
**Relative transcript levels of the *hmgR *genes of *M. circinelloides *under aerobic and anaerobic growth conditions.** MS12 was cultivated in liquid YNB under continuous light for 4 days at 25°C; transcript level of each gene measured under aerobic growth was taken as 1. The presented values are averages of three independent experiments; error bars indicate standard deviation. Relative transcript values followed by * significantly differed from the value taken as 1 according to the paired *t*-test (p < 0.01). Light micrographs show the morphology of the fungus growing under aerobic **(a)** and anaerobic conditions **(b)** and after 1-h incubation in the presence of oxygen after growing anaerobically for 4 days **(c)**; scale bars indicate 20 μm.

Morphological dimorphism is a characteristic feature of *M. circinelloides*. In the absence of oxygen and/or at high hexose concentration in the medium, filamentous growth of the fungus switches to a yeast-like form (Figure 5a, b). After growing for 4 days under anaerobic condition, relative transcript level of *hmgR1* significantly decreased, while the transcript concentration of *hmgR3* was found to be more than three times higher than that observed during aerobic growth (Figure 5). At the same time, transcript levels of *hmgR2* measured after aerobic and anaerobic growth did not differ significantly. If the anaerobic condition was ceased by placing the cultures in the presence of oxygen for 1 hour to induce the shift from the yeast-like morphology to the mycelial form (Figure 5c), relative transcript levels of all genes increased, and this increment was the most explicit in the case of *hmgR1* and *hmgR2*. If the transcript levels of the genes were correlated to that of the *hmgR1* at aerobic growth, *hmgR2* and *hmgR3* had the highest transcript level during aerobic and anaerobic growth, respectively (Additional file 1: Figure S6).

### Elevation of the copy number of the *hmgR* genes

*M. circinelloides* protoplasts were transformed with the circular plasmids, pNG1, pNG2 and pNG3 containing *hmgR1*, *hmgR2* and *hmgR3*, respectively (Additional file 1: Figure S8). Transformation frequencies were 8, 8 and 6 transformant colonies per 10^5^ protoplasts for pNG1, pNG2 and pNG3, respectively. PCR analysis of the transformants demonstrated that all of them contained the transferred plasmids (Additional file 1: Figure S9).

**Figure 6:**
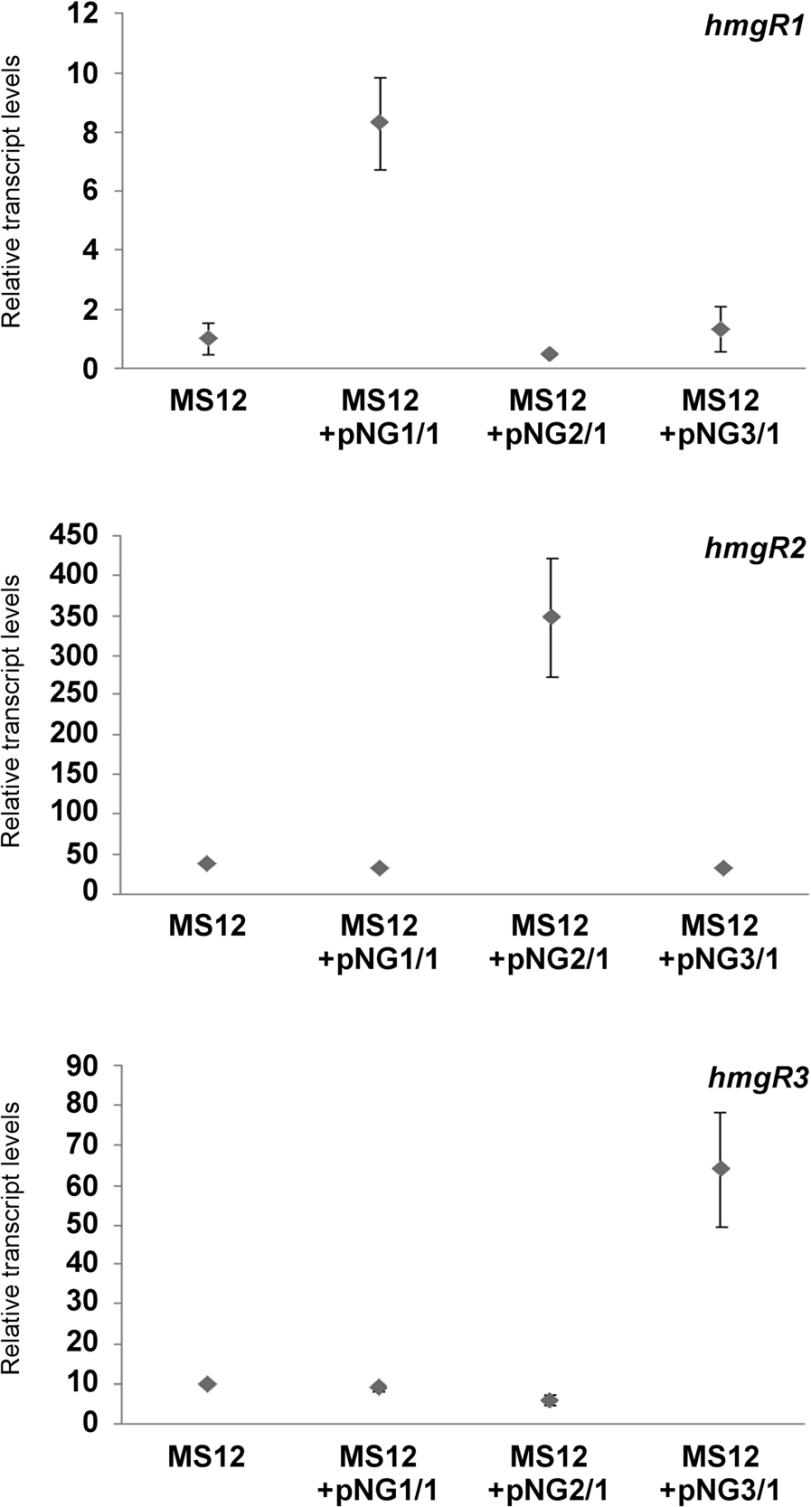
**Relative transcript levels of the *hmgR *genes of *M. circinelloides *measured in the transformants and the original MS12 strain.** MS12 was grown on YNB under continuous light for 2 days at 25°C; transcript level of each gene measured in the MS12 strain was taken as 1. The presented values are averages of three independent experiments; error bars indicate standard deviation.

Copy number of the transferred plasmids slightly fluctuated in the transformants during the consecutive cultivation cycles (Table 1). Genes introduced into the transformants in extra copies displayed elevated relative transcript levels, while the transcript concentration of the other isoforms did not change significantly (Figure 6). Total carotenoid content increased in the transformants harbouring pNG2 and pNG3, while the ergosterol content only changed slightly in the transformants that contained *hmgR2* in extra copies (Table 1).

**Table 1:** **Copy number of the transferred plasmids and total carotenoid and ergosterol content of the transformants in comparison with the original *M. circinelloides *** **strain**

	**Copy number of the plasmid**^**a**^	**Total carotenoid content (μg/g [dry weight] ± standard deviations)**	**Total ergosterol content (mg/g [dry weight] ± standard deviations)**
MS12	-	476 ± 56	6.0 ± 0.8
MS12 + pNG1	2-4	460 ± 68	5.9 ± 1.1
MS12 + pNG2	7-8	741 ± 86*	7.4 ± 0.8
MS12 + pNG3	1-4	846 ± 62*	6.5 ± 1.1

^a^Relative copy number means plasmid copies per the host genome.

Copy number of the plasmids as well as carotenoid and ergosterol content were measured in three independent transformants. In each case, carotenoid and ergosterol extractions and DNA purification were carried out from the same mycelial samples after cultivating the strains on YNB for 4 days under continuous light. Carotenoid and ergosterol contents are presented as averages ± standard deviation. Values followed by * significantly differed from the corresponding value of the MS12 strain according to the two-sample *t*-test (p < 0.01).

To test the sensitivity of the different transformants and MS12 to statins, MIC_90_ (minimum inhibitory concentration required to achieve 90% growth inhibition) values of fluvastatin, atorvastatin and rosuvastatin were determined for these strains (Table 2). Sensitivity to statins decreased in the transformants containing pNG2 and pNG3 possibly due to the gene dose effect.

**Table 2:** **Minimal inhibition concentration (MIC**_**90**_**) of three statins against the transformants and the original *M. circinelloides *** **strain**

**Strains/Statins**	**Fluvastatin (μg/ml)**	**Atorvastatin (μg/ml)**	**Rosuvastatin (μg/ml)**
MS12	4	16	32
MS12 + pNG1	4	16	32
MS12 + pNG2	32	256	>256
MS12 + pNG3	64	>256	>256

MIC_90_ values were determined for all transformants obtained from the different transformation experiments.

## Discussion

In this study, transcript levels of three HMG-CoA reductase genes of the carotene producing zygomycete fungus *Mucor circinelloides* were examined. This enzyme may be encoded by various numbers of isogenes in the different organisms. Animals generally have one *hmgR* gene; by contrasts, plant genomes may contain multiple genes [11]. Among fungi, one *hmgR* gene was found in *Schizosaccharomyces pombe*[14], while two isogenes were described in *Saccharomyces cerevisiae*[15]. Zygomycetes may contain one or more HMG-CoA reductases. In *Absidia glauca*, *B. trispora*, *P. blakesleeanus* and *Rhizomucor miehei*, only one gene has been detected [10,13,16] while the genome of *Rhizopus oryzae* contains two putative *hmgR* genes [10]; earlier, Burmester and Czempinksi [16] also reported two genes in *M. mucedo* and *Parasitella parasitica*. It can be suggested that a gene duplication event occurred in an ancestor of the zygomycetes and one of the genes was lost in certain lineages, while subsequent duplications occurred in other lineages during the divergence of this fungal group [10,13]. However, expression and regulation of these genes in zygomycetes have not yet been studied. In our tests, presence of the transcripts was proven for all three genes of *M. circinelloides* and they responded differentially to the changes in the tested cultivation conditions.

In case of *hmgR2* and *hmgR3*, patterns of the relative transcript levels differed from that of *hmgR1* during the cultivation of the fungus. Transcripts of *hmgR1* could be detected only in the mycelium (especially after 2 days postinoculation), while those of *hmgR2* and *hmgR3* are already present in the germinating spores and achieve high abundance in the young hyphae, i.e. at 8 h postinoculation (Figure 1). Although it is suggested that HMG-CoA reductase and mevalonate pathway affect fungal morphogenesis and polarized growth [10,17,18], there are very limited information on their role in the germination of spores and development of hyphae. In an early study, Basson et al. [15] reported that *S. cerevisiae* cells containing mutant alleles of both *HMG1* and *HMG2* were unable to undergo spore germination and vegetative growth.

It is known that membrane fluidity and structure, which are affected by the activity of HMG-CoA reductase through its role in the ergosterol biosynthesis, are important factors of the adaptation to the changing temperature of the environment. At the same time, cultivation temperature also affects the carotenoid production of *Mucor*[19-21]. Therefore, we examined the effect of the temperature on the transcript levels of the *hmgR* genes. The three genes responded differently to the temperature changes (Figure 2). Especially, *hmgR1* showed a temperature dependent transcript abundance, where increasing temperatures caused decreasing relative transcript levels. In case of *hmgR2* and *hmgR3*, such temperature dependence in the relative transcript levels was not observed.

Previously, HmgR activity and protein level were found to be essential for the adaptation of certain fungi to the changing salinity of the environment [8,22]. Thus, we examined whether salt stress has any effect on the transcription of the *hmgR* genes of *M. circinelloides*. Presence of 10 g/l NaCl in the medium upregulated all three genes, moreover concentration of *hmgR2* transcripts increased proportionally with the increasing salt concentrations (Figure 3). Modification of the amount and the composition of sterols in the cell membrane is an important factor of the adaptation to the osmotic changes of the environment [23]. Previously, HMG-CoA reductase was found to be regulated by environmental salinity in both salt-sensitive and salt-tolerant yeasts [8]. In that study, protein level and activity of HMG-CoA reductase in the salt-sensitive *S. cerevisiae* and the moderately halotolerant *A. pullulans* increased when these fungi were grown under high salt concentrations.

No data have been found about the effect of different carbon sources on the expression of *hmgR* genes. Therefore, transcription of these genes was examined after cultivating the fungus on five different carbon sources (Figure 4) found to stimulate the carotenoid production [19,20]. All three genes showed high relative transcript levels on sodium acetate and dihydroxyacetone. These compounds take part in the glycolysis and thus, may provide precursors for the mevalonate pathway. Acetate was found to affect terpenoid biosynthesis in *Blakesleea* and *Phycomyces*[1] and stimulated the HmgR enzyme activity in *Borrelia burgdorferi*[24]. In case of *hmgR2* and *hmgR3*, high transcript levels were detected on glucose also, which is known to be a good carbon source for terpenoid biosynthesis and carotenoid production in *Mucor*[20,21].

Compared to the transcript levels detected under aerobic growth, transcript levels of *hmgR1* and *hmgR3* changed oppositely under anaerobiosis, while that of *hmgR2* did not altered significantly (Figure 5). If the relative transcript levels of the genes were compared (i.e. if they were presented relative to one sample for a certain condition), *hmgR2* had the highest transcript level under aerobic cultivation condition during the whole life cycle of the fungus (Additional file 1: Figure S2). At the same time, transcripts of *hmgR3* had a moderate relative abundance under aerobic conditions but the gene displayed the highest relative transcript level compared to the other two genes under anaerobiosis (Additional file 1: Figure S6). These results indicate that oxygen level of the environment regulates the *hmgR* genes of *Mucor*. It is well known that the biosynthesis of ergosterol has high oxygen demand and regulatory effect of low-oxygen conditions on the transcription of the *hmgR* genes has also been proven in *S. cerevisiae*, *Aspergillus fumigatus*, *Cryptococcus neoformans* and *S. pombe*[9]. Somewhat similarly to the situation detected in *Mucor*, availability of oxygen affects the two *hmgR* genes of *S. cerevisiae* in an opposite manner: *HMG1* shows a stable and strong expression during aerobic growth while *HMG2* transcription is repressed; on the contrary, transcription of *HMG1* decreases and that of *HMG2* increases radically when oxygen is depleted [5,25].

In spite of the increased transcript level of *hmgR3*, the whole average ergosterol content radically decreased in the anaerobically grown cells (0.2 mg/g [dry weight]) compared to that of the aerobically grown mycelium (5.5 mg/g [dry weight]). Similar situation was previously observed in *M. genevensis*[26], which had <0.3 and 3 mg/g [dry weight] ergosterol content during anaerobic and aerobic growth, respectively.

In the transformation experiments, copy number of the *hmgR* genes was increased to overexpress them via the gene dose effect and carotenoid and ergosterol content as well as sensitivity to statins were analysed in the transformants. As carotenoids are synthesized through the mevalonate pathway, HMG-CoA reductase may affect the carotenoid level and manipulation of the encoding genes has been successfully used to increase the carotenoid content in some organisms [4,27,28]. It is known that statins are competitive inhibitors of HMG-CoA reductase and their antifungal effect, especially against opportunistic human pathogenic fungi, is an intensively studied area [29-31]. In several studies, zygomycetes were proven to be sensitive to these drugs [31-33] and lovastatin was found to induce apoptosis-like process in *Mucor*[6]. Previously, the *hmgR* gene of the closely related *R. miehei* was expressed in *M. circinelloides*[10]. In that study, expression of the exogenous *hmgR* did not affect the carotenoid content of *Mucor* but improved its resistance to fluvastatin. Overexpression of both *hmgR2* and *hmgR3* raised the carotenoid content (Table 1) and the resistance of the fungus to statins (Table 2). These results indicate that the product of the two genes may participate in the same processes or have overlapping functions but their regulations are different, as discussed above. Elevation of the copy number of *hmgR1* only moderately increased the relative transcript level of the gene and did not affect any tested feature*.* To decide whether *hmgR1* has any specific role in the cell needs further studies.

## Conclusions

In this study, transcription of the three HMG-CoA reductase genes of the β-carotene producing model organism, *M. circinelloides*, was analysed. Transcripts could be detected for all three genes but their transcription seems to be under different regulations suggesting functional differences among them. They seem to be especially involved in the adaptation to the changing oxygen tension and osmotic conditions of the environment as well as to statin treatment; overexpression of *hmgR2* and *hmgR3* significantly affected the carotenoid content. As the enzymes encoded by these genes have central roles in the terpene biosynthesis, exploration of their function and regulation is useful for further basic studies, e.g. to clarify the background of such important processes as fungal morphogenesis or adaptation to changing environment and in later applied research, e.g to improve the biosynthesis of carotenoids and other terpenoid compounds.

## Methods

### Strains, media and growth conditions

*M. circinelloides* strain MS12 (*leuA* and *pyrG*) [33] was used in the study; this strain is auxotrophic for leucine and uracil but wild-type for the carotene biosynthesis. *Escherichia coli* strain DH5α was used in all cloning experiments and plasmid amplifications. For nucleic acid, carotenoid and ergosterol extraction from *Mucor*, 10^6^ sporangiospores were plated onto solid minimal medium (YNB; 10 g glucose, 0.5 g yeast nitrogen base without amino acids (Difco), 1.5 g (NH_4_)_2_SO_4_, 1.5 g sodium glutamate and 20 g agar per litre) supplemented with leucine and/or uracil (0.5 mg/ml) if required. In some cases, RNA extraction was performed after cultivation in 30 ml YNB without agar; the inoculum size was 10^4^ sporangiospores/ml. Fungal cultures were grown for 4 days under continuous light at 25°C. Temperature dependence of the transcription was tested cultivating the fungal strains on solid YNB at 20, 25, 30 and 35°C. To examine the effect of different carbon sources on the gene expression, glucose was replaced with the tested compound in a final concentration of 10 g/l in YNB. When the effect of the different salt concentrations was analysed, NaCl were added to solid YNB to the final concentrations of 10, 20 and 30 g/l. To test the effect of fluvastatin on the gene expression, strains were grown in 30 ml liquid YNB containing fluvastatin in final concentrations of 1, 2 and 4 μg/ml. Anaerobic growth was performed in 30 ml liquid YNB in a BBL GasPak Anaerobic System (Becton Dickinson) at 25°C.

### Molecular techniques and cloning

General procedures for plasmid DNA preparation, cloning and transformation of *E. coli* were performed by following standard methods [34]. Genomic DNA and total RNA samples were prepared from mycelia disrupted with a pestle and mortar in liquid nitrogen. DNA was isolated using the MasterPure Yeast DNA Purification Kit (Epicentre), while RNA was purified using the E.Z.N.A Total RNA Kit II (Omega Bio-Tek). The genes were amplified by PCR using the Long PCR Kit (Thermo Scientific) and cloned into pBluescript II SK (Stratagene). Primers used to amplify the genes are presented in Additional file 1: Table S1.

### Sequence analysis of the putative HmgR proteins

Programs used to analyse the amino acid sequences of the putative HmgR proteins were accessed through the Swiss Expasy Server (http://www.expasy.ch). Molecular mass and pI of the proposed proteins were calculated by ProtParam [35] and the programs HMMTOP [36] and TMPred [37] were used for the transmembrane domain prediction. Domain search and prediction were performed using the Motif Scan (MyHits) program [38].

### Construction of plasmids and transformation

The isolated *hmgR* genes were placed between the promoter and terminator regions of the glyceraldehyde-3-phosphate dehydrogenase 1 gene (*gpd1*P and *gpd1*T, respectively) of *M. circinelloides* to assure their strong expression. The *hmgR1* gene was fused with *gpd1*P and *gpd1*T at the restriction sites *Sal*I and *Not*I of the plasmid pPT43 [38] arising pPT43-hmgR1, while *hmgR2* and *hmgR3* were ligated at the sites *Pst*I and *Not*I of pPT43 constructing pPT43-hmgR2 and pPT43-hmgR3, respectively. The *pyrG* gene of *M. circinelloides* was used as a selection marker in all constructed plasmids. It was cut from pPT81 [39] by the enzymes *Sca*I and *Kpn*I and was ligated into the same restriction sites of pPT43-hmgR1, pPT43-hmgR2 and pPT43-hmgR3, to create pNG1, pNG2 and pNG3, respectively (Additional file 1: Figure S8). PEG mediated protoplast transformations were performed according to van Heeswijck and Roncero [40]. Protoplasts were prepared as described earlier [41]. Transformants were selected on solid YNB medium based on the complementation of the uracil auxotrophy of the MS12 strain.

### qPCR analysis

Copy number and transcript level of the examined genes were analysed by using the real-time qPCR technique. Reverse transcription reactions were carried out with the Maxima H Minus First Strand cDNA Synthesis Kit (Thermo Scientific) using random hexamer and oligo(dT)18 primers, following the instructions of the manufacturer. The qPCR experiments were performed in a CFX96 real-time PCR detection system (Bio-Rad) using the Maxima SYBR Green qPCR Master Mix (Thermo Scientific) and the primers presented in Additional file 1: Table S1. The relative quantification of the copy number and the gene expression was achieved with the 2^-ΔΔCt^ method [42] using the actin gene (scaffold_07: 2052804-2054242) of *M. circinelloides* as a reference. In all experiments, reverse transcription was performed from the same RNA extract for each *hmgR* and the actin genes. Experiments were performed in biological and technical triplicates.

### Analysis of the carotenoid and the ergosterol contents

Carotenoids were extracted as described earlier [39]. Dried samples were dissolved in petroleum ether and their total carotenoid contents were analysed by spectrophotometry at 450 nm according to Rodriguez-Amaya [43]. Ergosterol extraction was performed using the method described by Alcazar-Fuoli et al. [44] with modifications. Three ml 25% alcoholic (methanol/ethanol, 3:2, v/v) KOH solution was added to the dried mycelia and the samples were vortexed for 3 min. After incubation at 85°C for 1 h, ergosterol was extracted with a mixture of 1 ml distilled water and 3 ml hexane followed by vigorous vortexing for 3 min. Extracts were centrifuged for 10 min at 1.900×g. The upper layer was transferred to a clean glass tube and the liquid phase was evaporated under nitrogen gas. For HPLC, a modular Shimadzu system equipped with a two channel UV/VIS detector was used. Dried samples were redissolved in 1 ml methanol, of which 50 μl was subjected on a Prodigy C18 (4.6×250 mm, ODS 5 μm) column (Phenomenex). Isocratic separation was performed with H_2_O/methanol (2:98, v/v) as mobile phase at a flow rate of 1.2 ml/min. The detection wavelengths were 210 and 280 nm; ergosterol standard was purchased from Sigma.

### Susceptibility tests

Sensitivity of the fungal strains to statins was examined in a 96-well microtiter plate assay. Fluvastatin (Lescol, Novartis), rosuvastatin (Crestor, Astra Zeneca) and atorvastatin (Atoris, Krka) were dissolved in methanol to prepare stock solutions. Final concentrations of fluvastatin in the wells ranged from 0.125 to 128 μg/ml while those of rosuvastatin and atorvastatin ranged from 0.5 to 256 μg/ml; statins were diluted with liquid YNB medium. Inocula were prepared and diluted in liquid YNB. The final amount of the sporangiospores in the wells was 10^4^. Plates were incubated for 48 h at 25°C and the optical density of the fungal cultures was measured at 620 nm using a Jupiter HD plate reader (ASYS Hitech). Uninoculated medium was used as the background for the calibration and growth in the statin-free medium was considered as 100%; all experiments were performed in triplicates.

### Availability of supporting data

All supporting data are included in the additional file.

## Competing interests

The authors declare that they have no competing interests.

## Authors’ contributions

GN: carried out most of the experimental work, performed the statistical analysis, and participated in the evaluation of the results and drafting the manuscript. AF and AC participated in the qPCR studies and the genetic transformation experiments. AS and OB measured the carotenoid and ergosterol contents. IN participated in the susceptibility tests and the analysis of the transformants. CV participated in the experimental design and was involved in drafting the manuscript. TP designed and evaluated all the experimental studies, participated in the sequence analysis and the genetic transformation studies and drafted the manuscript. All authors read and approved the final manuscript.

## Supplementary Material

Additional file 1: Table S1 Primers used in the present study. **Table S2.** Main features of the three HmgR proteins. **Figure S1.** Amino acid sequence of the three HMG-CoA reductases of *Mucor circinelloides* aligned to other known HmgR proteins. **Figure S2.** Relative transcript levels of the *M. circinelloides hmgR* genes during the cultivation period. Relative transcript level of *hmgR1* at 96 hours was taken as 1. **Figure S3.** Relative transcript levels of the *M. circinelloides hmgR* genes at different cultivation temperatures. Relative transcript level of *hmgR1* at 25°C was taken as 1. **Figure S4.** Relative transcript levels of the *M. circinelloides hmgR* genes at different salt concentrations. Relative transcript level of *hmgR1* of the untreated control was taken as 1. **Figure S5.** Relative transcript levels of the *hmgR* genes of *M. circinelloides* growing on different carbon sources. Relative transcript level of *hmgR1* on YNB with glucose was taken as 1. Hyphal morphology on the different carbon sources are showed on the light micrographs. **Figure S6.** Relative transcript levels of the *M. circinelloides hmgR* genes under aerobic and anaerobic growth conditions. Relative transcript level of *hmgR1* when the fungus was grown under aerobic condition was taken as 1. Morphology of MS12 under aerobic and anaerobic conditions are showed on the light micrographs. **Figure S7.** Reverse transcription - PCR of the investigated genes. PCR conditions and primers were the same as in the qPCR experiments. **Figure S8.** Maps of the plasmids used in this study. **Figure S9.** PCR amplification of the transferred plasmids from the *M. circinelloides* transformants. Primers used in these experiments were designed to the terminus of the *gpd*P (Gpdp) and the first part of each *hmgR* gene. Sequences of the primers are shown in **Table S1.** Click here for file
