# Supplementary material for: Transcription of the three HMG-CoA reductase genes of Mucor circinelloides
Source: BMC Microbiol. 2014 Apr 14;14:93. doi: 10.1186/1471-2180-14-93 (PMC4037427; doi:10.1186/1471-2180-14-93)
Supplement: Additional file 1: Table S1 — Primers used in the present study. Table S2. Main features of the three HmgR proteins. Figure S1. Amino acid sequence of the three HMG-CoA reductases of Mucor circinelloides aligned to other known HmgR proteins. Figure S2. Relative transcript levels of the M. circinelloides hmgR genes during the cultivation period. Relative transcript level of hmgR1 at 96 hours was taken as 1. Figure S3. Relative transcript levels of the M. circinelloides hmgR genes at different cultivation temperatures. Relative transcript level of hmgR1 at 25°C was taken as 1. Figure S4. Relative transcript levels of the M. circinelloides hmgR genes at different salt concentrations. Relative transcript level of hmgR1 of the untreated control was taken as 1. Figure S5. Relative transcript levels of the hmgR genes of M. circinelloides growing on different carbon sources. Relative transcript level of hmgR1 on YNB with glucose was taken as 1. Hyphal morphology on the different carbon sources are showed on the light micrographs. Figure S6. Relative transcript levels of the M. circinelloides hmgR genes under aerobic and anaerobic growth conditions. Relative transcript level of hmgR1 when the fungus was grown under aerobic condition was taken as 1. Morphology of MS12 under aerobic and anaerobic conditions are showed on the light micrographs. Figure S7. Reverse transcription - PCR of the investigated genes. PCR conditions and primers were the same as in the qPCR experiments. Figure S8. Maps of the plasmids used in this study. Figure S9. PCR amplification of the transferred plasmids from the M. circinelloides transformants. Primers used in these experiments were designed to the terminus of the gpdP (Gpdp) and the first part of each hmgR gene. Sequences of the primers are shown in Table S1. [file 1471-2180-14-93-S1.pdf]

Additional file 1

Transcription analysis of the three HMG-CoA reductase genes of *Mucor circinelloides*

By: Gábor Nagy, Anita Farkas, Árpád Csernetics, Ottó Bencsik, András Szekeres, Ildikó Nyilasi, Csaba Vágvölgyi, Tamás Papp

Table S1 - Primers used in the present study.

Primer	Sequence 5’-3’	Amplified DNA
Primers used to amplify the *hmgR* genes		
Hmg-coA2f	GGCCTGCAGATGATCAATCTGCTGTCCTAT[a]	*hmgR1*
Hmg-coA2r	TTCGCGGCCGCCTATAAGATGCAATTTCCCGTT	
Hmg-coA3f	TTCGTCGAATGTTGAAAAACGTCAAAAAAGAT	*hmgR2*
Hmg-coA3r	TTCGCGGCCGCCTATGATTTAATACAACTTCCA	
Hmg-coA4f	TTCGTCGACATGGCGTCTGCTCTCCCCCATCA	*hmgR3*
Hmg-coA4r	TTCGCGGCCGCTTAGGATTTGATGCAGGTT	
Primers used in the qPCR experiments		
H1_RT_F	CAGAAACAACTCACATGCCTTGCC	151 bp of
H1_RT_R	TAATAGCTGGAAGTGGATCGTCGG	*hmgR1*
H2_RT_F	CTCGTATCATCTGTGCCTCTG	107 bp of
H2_RT_R	AGCAGTGTTACGGTTGTGAG	*hmgR2*
hmgR3_RTf	GAATCCAGTCTGTTGCCTCTCCA	128 bp of
hmgR3_RTr:	GAGTCACCGTCAATGTTCATGGGA	*hmgR3*
MCactinF	CACTCCTTCACTACCACCGCTGA	117 bp of
MCactinR	GAGAGCAGAGGATTGAGCAGCAG	actin
Primers used to analyse the transformants in the demonstration of plasmids		
Gpdp	CATGAAGTGTGAGACATTGCGA	
png1rev	TGTTGTCTCGATTGAATGGCTGG	
png2rev	TCCACAGGATTGGCGGTCTAT	
png3rev	CAGCGTGTGGTCAATGGTGTT	

[a] Underlined characters indicate the restriction enzyme sites designed for cloning of the amplicons.

Table S2 - Main features of the three HmgR proteins.

	HMGR1	HMGR2	HMGR3
Length	1107 aa	1078 aa	1115 aa
Calculated molecular mass	120.78 kDa	118.45 kDa	120.71 kDa
Calculated pI	8.86	8.33	8.43
Number of trans-membrane helices	6	9	5
Sterol-sensing domain	244-407 aa	231-383 aa	279-443 aa
Catalytic domain	653-1107 aa	628-1079 aa	692-1115 aa
HMG-binding motifs	CENVIGYMPIP (717-727 aa); PMATTEGCLVA (744-754 aa)	CENVIGYMPIP (692-702 aa); PMATTEGCLVA (719-729 aa)	CENVIGYMPIP (756-766 aa); PMATTEGCLIA (783-793 aa)
NAD(P)H-binding motifs	TGDAMGMN (840-847 aa); VGTVGGGT (990-997 aa)	TGDAMGMN (815-822 aa); VGTIGGGT (965-972 aa)	TGDAMGMN (879-886 aa); VGTIGGGT (1029-1036 aa)

```
                   10        20        30        40        50        60
          ....|....|....|....|....|....|....|....|....|....|....|....|
McHmgR1   -MINLLSYKSITGFMN------RLVLKTSKVSALNPIETMVASLILGSVTYVYLFNLAKS 53
McHmgR2   ---MLKNVKKDNPFR-----------YLAHVSARNPFEMMVAVFIACSFSYLYLTNTYNK 46
McHmgR3   MASALPHHSGTSAFRSISSVFGKGIHRAAKLSSRNPIEMIAGILILSSFSYFYLFNLART 60
RmHmgR    MPSSTRILGTSSAFRSYSALVGKGIHHAARLSTRNPIEMFVGVIILASFSYVCLYNLART 60
PbHmgR    --MSLPNHSGSSAFKSFSYIVGTGIKRAAKLSTRNPIEMIVVVLILSSFSYFYLFNLART 58
SpHmgR    -----MIYKLAARYPIQVIAIVGILVSMAYFSFLEALTQEDFPVLIRALKRFGILDGFPN 55
ScHMG1    -----MPPLFKG-----LKQMAKPIAYVSRFSAKRPIHIILFSLIISAFAYLSVIQYYFN 50
ScHMG2    -----MSLPLKT-----IVHLVKPFACTARFSARYPIHVIVVAVLLSAAAYLSVTQSYLN 50
MaHMGR    --------MLSRLFR-----------MHGLFVASHPWEVIVGTVTLTIC----------- 30
HsHMGR    --------MLSRLFR-----------MHGLFVASHPWEVIVGTVTLTIC----------- 30

                   70        80        90       100       110       120
          ....|....|....|....|....|....|....|....|....|....|....|....|
McHmgR1   SEILSTASIYDTSFVSTILYASPNDISFSPLKQ----DPPLPAVSISRIELKQIAIALSD 109
McHmgR2   G---LHDMTS---THPIAYYHSNQSTHHHGNSN----------DDNKSVILKQLVLH-HR 89
McHmgR3   SDIFSGTVTR---LYPTSVYADKHTQGFQQLSRNDASLDTTTTEEAVKIQLRQISIVDQE 117
RmHmgR    SDIFSGTNMR---LYPATVYAPSNGLEFSPVDS------SFVDKTAQKIHLRQITVTAEE 111
PbHmgR    SDIFSGTVTR---LYPTSVYAPTKDHSFSVVDR---TADSTIANNAVKVHLHQIVVSDPK 112
SpHmgR    TRLPNEMI------LKLSSVQGEDASVWEQIPAAELGGEGFVDFDITQWYYPANAKVDVA 109
ScHMG1    GWQLDSNS------VFETAPNKDSNTLFQECS------HYYRDSSLDGWVSIT--AHEAS 96
ScHMG2    EWKLDSN-------QYSTYLSIKPDELFEKCT------HYYRSPVSDTWKLLS--SKEAA 95
MaHMGR    -------------MMSMNMFTGNNKICGWNYECP-------------------------- 51
HsHMGR    -------------MMSMNMFTGNNKICGWNYECP-------------------------- 51

                  130       140       150       160       170       180
          ....|....|....|....|....|....|....|....|....|....|....|....|
McHmgR1   P--AHQHNMDAIQRFRNHVESTPVDFSDLEHQEKGLMTYKDHLCYNA------------- 154
McHmgR2   QDMMTKEALQSVLSFQESLAQ---DISE--------------FCYRN------------- 119
McHmgR3   KNVIDRNTLATILRFQNTIDH---TLLD---DHVGQFGYS-ALCFKN------------- 157
RmHmgR    L----QDHLSSVERFQKYLEN---DVYVP--DGTRQFSYSKGLCYST------------- 149
PbHmgR    HGVLSRQTLASVLRFQQMAEN---EIYVPDSTAVNRFAFNKDLCYKTTLPSSYSSHSSDS 169
SpHmgR    QLVEPYRNDCIFHDASGACHF--------------------------------------- 130
ScHMG1    ELPAPHHYYLLNLNFNSPNET--------------------------------------- 117
ScHMG2    DIYTPFHYYLSTISFQSKDNS--------------------------------------- 116
MaHMGR    -------------KFEEDVLS--------------------------------------- 59
HsHMGR    -------------KFEEDVLS--------------------------------------- 59

                  190       200       210       220       230       240
          ....|....|....|....|....|....|....|....|....|....|....|....|
McHmgR1   ---------STTCLTCQP---------------------------------------FNR 166
McHmgR2   --------EDNDCVIKSPLNFWNNSIAKLNADRNVKSTIYQQQQQHDQSHYFLMNHHNNY 171
McHmgR3   --------AQGECFSQSLANIFDMDT--IDSDD--LRRSINQRPELAASIFGELDLNASS 205
RmHmgR    ---------NENCFSAS---------------------------------------TDKD 161
PbHmgR    NSNSNLNSKTSPCFAHSPADIWQDEATLLADKN--IRSTIEANLDTAKNVFGDLQLNATY 227
SpHmgR    ------------------------------------------------------------ 130
ScHMG1    ------------------------------------------------------------ 117
ScHMG2    ------------------------------------------------------------ 116
MaHMGR    ------------------------------------------------------------ 59
HsHMGR    ------------------------------------------------------------ 59

                  250       260       270       280       290       300
          ....|....|....|....|....|....|....|....|....|....|....|....|
McHmgR1   DNSAILSYVFDLSNDQRIKASHLWDQKVMSSSVDRLIPMATQHQYNERSISTVVWLIRIM 226
McHmgR2   ADAVVLSFVFDGS---QQDRVNQWEHNIAKLGP----IFSHQSKQP----NYMTMIFDHA 220
McHmgR3   ASSILLSFAFNASTEYRQQLSYAWEQKVSTLSSGDLVSLSNTGHQE----DVFTWLFIIT 261
RmHmgR    TSILTLSFAFDASNAMRRDLANQWENKVAKLPPGELVSQAQQGYGD----NLLVWFFIIT 217
PbHmgR    ASSVTLSYAFNTTGDYREHLADMWKHKVATLPPADLVSLSNIGQQE----NVFAWLFIVT 283
SpHmgR    FFKEVGNWTVSSIALPSNLANPPIDYFLDSSSTVIQRILPAIREHG----ISWSWLLQLI 186
ScHMG1    DSIPELANTVFEKDNTKYILQEDLSVSKEISSTDGTKWR--LRSDR----KSLFDVKTLA 171
```

```
ScHMG2   TTLPSLDDVIYSVDHTRYLLSEEPKIPTELVSENGTKWR--LRNNS----NFILDLHNIY 170
MaHMGR   ------------------------------------------------------------ 59
HsHMGR   ------------------------------------------------------------ 59

                     310       320       330       340       350       360
         ....|....|....|....|....|....|....|....|....|....|....|....|
McHmgR1  KNIARDTAVRMNSASKMDVIVVTAGYIMMAFTFLSLYIRMH-KLGSKYTLATTIFMSGFF 285
McHmgR2  -------KRLYKTANPVDLLLLTSGYVFMFITLINLFYSLRKVVGSYCIIGSAVILNGIC 273
McHmgR3  RNIVYRIKELIDMADNIDIIVILGGYMMMLTTFVSLYMNMR-SMGSRYTLATAVVVNGFF 320
RmHmgR   RNVVFRLKELIEMADNVDIIVILAGYIMMIATFVSLYINMR-RMGSRYTLATAVTVNGLF 276
PbHmgR   RNVIFRVKELIDLADNIDIIVILVGYIMMIATFISLYVNMR-AMGSRYTLATAVVFNGFF 342
SpHmgR   ARTWMNTLKIASQASKTELLIVGTAYACMLISIVSLYLKMR-RLGSKFWLFFSVLLSTLF 245
ScHMG1   YSLYDVFSENVTQADPFDVLIMVTAYLMMFYTIFGLFNDMR-KTGSNFWLSASTVVNSAS 230
ScHMG2   RNMVKQFSNKTSEFDQFDLFIILAAYLTLFYTLCCLFNDMR-KIGSKFWLSFSALSNSAC 229
MaHMGR   ----------------SDIIILTITRCIAILYIYFQFQNLR-QLGSKYILGIAGLFTIFS 102
HsHMGR   ----------------SDIIILTITRCIAILYIYFQFQNLR-QLGSKYILGIAGLFTIFS 102

                     370       380       390       400       410       420
         ....|....|....|....|....|....|....|....|....|....|....|....|
McHmgR1  SFMLSLATVY-KLGAPVSPVLLSEATPFLVVTIGFERPYKLTKCIFE-QESNN-TDNSNV 342
McHmgR2  ALVFGLTAMK-LLHVNITPALLLESLPFLVITIGFGRHIKYTKSILS-NTNNN------- 324
McHmgR3  SFMFALLTVN-ALGVDVYPVVLAEAIPFLAVTIGFERHFKLTKRVFE-FSKETPLTKQEI 378
RmHmgR   SFMLALLTVH-ALGVEPSPVVLAEAIPFLVVTVGFERPYKLTKRVFE-YSKEAPITRQDV 334
PbHmgR   SFMLALLTVR-ALGVDVYPVVLAEAIPFLAVTIGFERPFKLTKRVFQ-FSKETPLTKQEI 400
SpHmgR   SVQFAMTLVR-ASGVRISLVSLIESLPFLINVVALDKAAELTRQVITRCSVS--DSHSPM 302
ScHMG1   SLFLALYVTQCILGKEVSALTLFEGLPFIVVVVGFKHKIKIAQYALEKFERVGLSKRITT 290
ScHMG2   ALYLSLYTTHSLLKKPASLLSLVIGLPFIVVIIGFKHKVRLAAFSLQKFHRISIDKKITV 289
MaHMGR   SFVFSTVVIH-FLDKELT--GLNEALPFFLLLIDLSRASALAKFALS-SNSQD-----EV 153
HsHMGR   SFVFSTVVIH-FLDKELT--GLNEALPFFLLLIDLSRASTLAKFALS-SNSQD-----EV 153

                     430       440       450       460       470       480
         ....|....|....|....|....|....|....|....|....|....|....|....|
McHmgR1  LQIISRSVEMMAPTLIRDGLLEIVVFILG--AKSTLPGLREFCLMSAFLIAYDMMLMFTW 400
McHmgR2  ------NDNSVFMMLAWDCFTEITIFCLG--AKSGISGLREFCWLSACLLAFDFVLLCTW 376
McHmgR3  RKTIVRAVDSVALPIARDCIMEITVLALG--AKSGISGLREFCLLSAILLAYDFIIMFTW 436
RmHmgR   RETIMRAVDAVALPIARDCVIEIMVLGLG--AKSAIVGLREFCVLSAFLLAYDVVFLFTW 392
PbHmgR   RTTIMRAVDTVALPIARDCFMEIIVLVLG--AKSGISGLEEFCLLSAILLAYDFIIMFTW 458
SpHmgR   HEDIAKACRNAAPPILRHFSFGIVVLA---IFSYCNFGIKQFFLFAA-VMIYDLLLLFSF 358
ScHMG1   DEIVFESVSEEGGRLIQDHLLCIFAFIGCSMYAHQLKTLTNFCILSAFILIFELILTPTF 350
ScHMG2   SNIIYEAMFQEGAYLIRDYLFYISSFIGCAIYARHLPGLVNFCILSTFMLVFDLLLSATF 349
MaHMGR   RENIARGMAILGPTFTLDALVECLVIGVG--TMSGVRQLEIMCCFGCMSVLANYFVFMTF 211
HsHMGR   RENIARGMAILGPTFTLDALVECLVIGVG--TMSGVRQLEIMCCFGCMSVLANYFVFMTF 211

                     490       500       510       520       530       540
         ....|....|....|....|....|....|....|....|....|....|....|....|
McHmgR1  YISVLSLKLELRKIKETTS-------TSTTTTTSILETAAATSQVASTSTTTINDSHH-- 451
McHmgR2  YTAILALKLEVIGLVFHT--------FGRGCFVCVMMIYIVLFLAESIQYGRRDQAPAKQ 428
McHmgR3  YTAVLALKLELLRIREINGTSN---KSAKTTGTG--YIRSTVVKAFSDSTTT-NNSSS-- 488
RmHmgR   YTAVLALKLELRRIREISGSSKASSQGGAPQPSGVSFIRKTVVKALSDDNAAGTTTTPQD 452
PbHmgR   YTAVLALKLELLRIREINGISADDIKKGTKKSTG--YIRRTVIKAFSDDHAAGANTAN-- 514
SpHmgR   FVAILTLKLEMRRYN-------------AKDDVRKVLIEEGLSESTARHVADGNDSSATT 405
ScHMG1   YSAILALRLEMNVIH-------------RSTIIKQTLEEDGVVPSTARIISKAEKKSVSS 397
ScHMG2   YSAILSMKLEINIIH-------------RSTVIRQTLEEDGVVPTTADIIYKDETASEPH 396
MaHMGR   FPACVSLVLELSRE------------SREGRPIWQLSHFARVLEEEENKPNPVTQRVK-- 257
HsHMGR   FPACVSLVLELSRE------------SREGRPIWQLSHFARVLEEEENKPNPVTQRVK-- 257

                     550       560       570       580       590       600
         ....|....|....|....|....|....|....|....|....|....|....|....|
McHmgR1  -ARNKKPAIIKTKLLMIIGFLTMHVFEFCSTLSFSTSRNNSHALPQVPVIGPTIEPVLSH 510
McHmgR2  LQQRICLALDPGKDEAIVGFIGMHIFKVCSTFQQEP-----LSFTTVDTSQPTVAFVLSE 483
McHmgR3  -IKTDEPIIGRVKLLMIVGFVAMQLFKVCTTFQG-------SSGPQVSVVEPGVNNVLEK 540
```

```
RmHmgR    MQKSDGFFIGHVKLLMIVAFVSMHIFEFCTAFQ---------SGPQVDVSQPAVSSVLSQ 503
PbHmgR    -QKADGPIIGRVKLLMIVGFVVMHIFKFCSAFQ--------SVGPQVNITEPSIAVVLDQ 565
SpHmgR    SAGSRYFKVRYGTKIILFIFIAFNLFELCSIPFKHY----AATSAAARLIPLVRSQYPD 461
ScHMG1    FLNLS-VVVIIMKLSVILLFVFINFYNFGAN---------WVNDAFNSLYFDKERVSLPD 447
ScHMG2    FLRSN-VAIILGKASVIGLLLLINLYVFTDK---------LNATILNTVYFDSTIYSLPN 446
MaHMGR    MIMSLGLVLVHAHSRWIADPSPQNSTTEHSKVSLG---LDEDVSKRIEPSVSLWQFYLSK 314
HsHMGR    MIMSLGLVLVHAHSRWIADPSPQNSTADTSKVSLG---LDENVSKRIEPSVSLWQFYLSK 314

                   610       620       630       640       650       660
          ....|....|....|....|....|....|....|....|....|....|....|....|
McHmgR1   ILK-------------QHRLSGSTNG-LLLKVSPTIH--FQLLAQTHFQRLP-PVKSLLA 553
McHmgR2   LLD-------------KQPLVKVFPNQYFYAVISPSATCFSTSSLFQQYQLYYIMIKPFE 530
McHmgR3   LLE-------------QHRASDMGHLPLLVEVFPPLP--FHIASSSYKAIVPDSIRQPLD 585
RmHmgR    LLQ-------------EHRASPHAAKPLLVEVFPPLL--FRVPSTN-NGVLPESISKTLD 547
PbHmgR    LLE-------------QHKASSQASLPLFVQVFPAMP--FHVATVN-KSFVPDAITRPLE 609
SpHmgR    FKSQRLLDDGVFDDVLSAISSMSNIESPSVRLLP--------AVFYGAELSSTSFLSTIH 513
ScHMG1    FIT------------SNASENFK--EQAIVSVTP--------LLYYKPIKSYQRIEDMVL 485
ScHMG2    FIN------------YKDIGNLS--NQVIISVLP--------KQYYTPLKKYHQIEDSVL 484
MaHMGR    MIS-------------MDIEQVVTLSLAFLLAVKYIF--FEQAETESTLSLKNPITSPVV 359
HsHMGR    MIS-------------MDIEQVITLSLALLLAVKYIF--FEQTETESTLSLKNPITSPVV 359

                   670       680       690       700       710       720
          ....|....|....|....|....|....|....|....|....|....|....|....|
McHmgR1   EIYNTYA-VYAQDPVISKWVVAILMVSILLNTYLFEIAKYNR------------------ 594
McHmgR2   SLFDIYA-VYIQHPVISKWLTIALCVSLFLNTYLFNVAKQQP------------------ 571
McHmgR3   YLLETYA-VYIQHPVISKWITLALFVSLFLNTYLFKVAKQPA------------------ 626
RmHmgR    ALSEVYE-VYVQHPVISKWITIGLCVSLFLNTYLFNVAKQPK------------------ 588
PbHmgR    ALFDTYA-VYIQHPVISKWLTIALFVSLFLNTYLFNVAKQPK------------------ 650
SpHmgR    SFINNWS-HYISASFLSKWIVCALSLSIAVNVFLLNAARLNS------------------ 554
ScHMG1    LLLRNVS-VAIRDRFVSKLVLSALVCSAVINVYLLNAARIHTSYTADQLVKTEVTKKSFT 544
ScHMG2    LIIDSVS-NAIRDQFISKLLFFAFAVSISINVYLLNAAKIHTGYMNFQPQSNKID--DLV 541
MaHMGR    TPKKAPDNCCRREPLLVRRSEKLSSVEEEPGVSQDRKVEVIK------------------ 401
HsHMGR    TQKKVPDNCCRREPMLVRNNQKCDSVEEETGINRERKVEVIK------------------ 401

                   730       740       750       760       770       780
          ....|....|....|....|....|....|....|....|....|....|....|....|
McHmgR1   ---QTAQRNQPIQELRS----------------VPITSPR-------------------- 615
McHmgR2   ---KVVVQKVIEKVQVP--------------VQVPAAKKE-------------------- 594
McHmgR3   ---KAAPAAAAETKKS-------------TPTVVPTITT-------------AERKTQHH 657
RmHmgR    ---QISEKPEAETVVAP---------------PKPKVVAP-------------------- 610
PbHmgR    ---QIVEQVNQDKKITNAIESTNNTHIEVTEKQKPTIQSPGPVVSSAVVMSPNHKRSHNH 707
SpHmgR    ------IKEEPEKKVVEKVVE--------VVKYIPSSNSS-------------------- 580
ScHMG1    APVQKASTPVLTNKTVISGSK--------VKSLSSAQSSS-------------------- 576
ScHMG2    VQQKSATIEFSETRSMPASSG--------LETPVTAKDII-------------------- 573
MaHMGR    ---PLVVETESASRATFVLG-------------ASGTSPP-------------------- 425
HsHMGR    ---PLVAETDTPNRATFVVGN------------SSLLDTS-------------------- 426

                   790       800       810       820       830       840
          ....|....|....|....|....|....|....|....|....|....|....|....|
McHmgR1   ----LRSAISASS------PQPQLGMRTVEECLHILRDIDQGASHLADEEIVLLVQHAHI 665
McHmgR2   ----KKVAPASPS------HHRSDIVRPLDEVFSLIGTPEV----LTDEEIISVVQSGKM 640
McHmgR3   HHHQKHSSKHSA---------VQGVIRTLEECMQLTQTPES----LSDEEVIMLVQKGKM 704
RmHmgR    ------PPPPAP----------TGTIRSVDECLALIKTPEA----LNDEEIIQLVNAGKI 650
PbHmgR    HHSHSHSHNHHSN------HHQSDIVRPIDECVALVRTPEM----LNDEEVISLVENGKM 757
SpHmgR    ----SIDDIQKDE------IAQESVVRSLEECITLYNNGQIST--LNDEEVVQLTLAKKI 628
ScHMG1    ----SGPSSSSEEDDSRDIESLDKKIRPLEELEALLSSGNTKQ--LKNKEVAALVIHGKL 630
ScHMG2    ----ISEEIQNNE-CVYALSSQDEPIRPLSNLVELMEKEQLKN--MNNTEVSNLVVNGKL 626
MaHMGR    ----VAARTQELE------IELPSEPRPNEECLQILESAEKGAKFLSDAEIIQLVNAKHI 475
HsHMGR    ----SVLVTQEPE------IELPREPRPNEECLQILGNAEKGAKFLSDAEIIQLVNAKHI 476

                   850       860       870       880       890       900
```

```
        ....|....|....|....|....|....|....|....|....|....|....|....|
McHmgR1 APYALEKVLGDLERAVHIRKTVISRSSITQTLESSALPVAEYDYDKVLGACCENVIGYMP 725
McHmgR2 AAYALEKVLGDFERAVHIRRALVSRDSITKSLEGSLLPVKNYHYDKVMGACCENVIGYMP 700
McHmgR3 ASYALEKVLGDLERAVSIRRALVSRASITKTLESSLLPLHNYHYDKVMGACCENVIGYMP 764
RmHmgR  ASYALEKMLGDMQRAVSIRRALISRASVTRTLETSLLPLKDYHYEKVFGACCENVIGYMP 710
PbHmgR  ASYALEKVLGDLQRAVGIRRALISRASITKTLEASALPLENYHYDKVMGACCENVIGYMP 817
SpHmgR  PLYALERVLKDVTRAVVIRRTVVSRSSRTKTLESSNCPVYHYDYSRVLNACCENVIGYMP 688
ScHMG1  PLYALEKKLGDTTRAVAVRRKALSILAEAPVLASDRLPYKNYDYDRVFGACCENVIGYMP 690
ScHMG2  PLYSLEKKLEDTTRAVLVRRKALSTLAESPILVSEKLPFRNYDYDRVFGACCENVIGYMP 686
MaHMGR  PAYKLETLMETHERGVSIRRQLLS-TKLPEPSSLQYLPYRDYNYSLVMGACCENVIGYMP 534
HsHMGR  PAYKLETLMETHERGVSIRRQLLS-KKLSEPSSLQYLPYRDYNYSLVMGACCENVIGYMP 535

                      910       920       930       940       950       960
        ....|....|....|....|....|....|....|....|....|....|....|....|
McHmgR1 IPVGVAGPMMIDGESIHLPMATTEGCLVASVARGCKAVNVNG-ATTVLISDGMTRGPCVE 784
McHmgR2 IPVGVAGPLNIDGDLIHIPMATTEGCLVASAARGCKAINAGGGATTIVTADGMTRGPCVE 760
McHmgR3 IPVGVAGPMNIDGDSIHIPMATTEGCLIASAARGCKAINAGGGATTIITADGMTRGPCVE 824
RmHmgR  LPLGVAGPLNIDGIPTHIPMATTEGCLVASTARGCKAINAGGGATTIVTADGMTRGPCVE 770
PbHmgR  IPVGVAGPMNIDGDLIHIPMATTEGCLVASTARGCKAINAGGGASTIVIADGMTRGPCVE 877
SpHmgR  LPLGVAGPLIIDGKPFYIPMATTEGALVASTMRGCKAINAGGGAVTVLTRDQMSRGPCVA 748
ScHMG1  LPVGVIGPLVIDGTSYHIPMATTEGCLVASAMRGCKAINAGGGATTVLTKDGMTRGPVVR 750
ScHMG2  IPVGVIGPLIIDGTSYHIPMATTEGCLVASAMRGCKAINAGGGATTVLTKDGMTRGPVVR 746
MaHMGR  IPVGVAGPLCLDGKEYQVPMATTEGCLVASTNRGCRAIGLGGGASSRVLADGMTRGPVVR 594
HsHMGR  IPVGVAGPLCLDEKEFQVPMATTEGCLVASTNRGCRAIGLGGGASSRVLADGMTRGPVVR 595

                      970       980       990      1000      1010      1020
        ....|....|....|....|....|....|....|....|....|....|....|....|
McHmgR1 FPNIIDAGLCKRWLDQEEGFEIVAEAFNSTSRFARVRKMQVAMAGKLLYIRFSTTTGDAM 844
McHmgR2 FPDILRAADCKRWIEQE-GEKVVTEAFNSTSRFARVRKLKVALAGRLMYIRFSTTTGDAM 819
McHmgR3 FPSIIGAAACKKFIEED-GAEIITAAFNSTSRFARLRKLKVALAGRLVFIRFSTTTGDAM 883
RmHmgR  FPNITRAGECKRWIENE-GYEVIADAFNATSRFARLRKLKVTLAGKLVFIRFSTTTGDDM 829
PbHmgR  FPTILRAAACKLWIENE-GNDIVTNAFNSTSRFARLRKLKIALAGKLVFIRFSTTTGDAM 936
SpHmgR  FPDLTRAGRAKIWLDSPEGQEVMKKAFNSTSRFARLQHIKTALAGTRLFIRFCTSTGDAM 808
ScHMG1  FPTLKRSGACKIWLDSEEGQNAIKKAFNSTSRFARLQHIQTCLAGDLLFMRFRTTTGDAM 810
ScHMG2  FPTLIRSGACKIWLDSEEGQNSIKKAFNSTSRFARLQHIQTCLAGDLLFMRFRTTTGDAM 806
MaHMGR  LPRACDSAEVKAWLETPEGFAVIKDAFDSTSRFARLQKLHVTMAGRNLYIRFQSKTGDAM 654
HsHMGR  LPRACDSAEVKAWLETSEGFAVIKEAFDSTSRFARLQKLHTSIAGRNLYIRFQSRSGDAM 655

                     1030      1040      1050      1060      1070      1080
        ....|....|....|....|....|....|....|....|....|....|....|....|
McHmgR1 GMNMISKGCEKALSKIAEYF--PTMQIVSLSGNYCTDKKPAAINWIEGRGKSVVAEAVIP 902
McHmgR2 GMNMISKGCEKALSKIAERY--PDMQIISLSGNYCTDKKPAAINWIEGRGKSVVAEAVIP 877
McHmgR3 GMNMISKGCEKALSVLSEHF--PDMQIVSLSGNYCTDKKPAAINWIEGRGKSVVAEAVIP 941
RmHmgR  GMNMISKGCEAALSVLAEEF--PDMQIISLSGNYCTDKKPAVINWIEGRGKSVVAEAIIP 887
PbHmgR  GMNMISKGCEKALSIITEHF--PDMQIISLSGNYCTDKKPAAINWIEGRGKSVVTEAVIP 994
SpHmgR  GMNMISKGVEHALVVMSNDAGFDDMQVISVSGNYCTDKKPAAINWIDGRGKSVIAEAIIP 868
ScHMG1  GMNMISKGVEYSLKQMVEEYGWEDMEVVSVSGNYCTDKKPAAINWIEGRGKSVVAEATIP 870
ScHMG2  GMNMISKGVEYSLKQMVEEYGWEDMEVVSVSGNYCTDKKPAAINWIEGRGKSVVAEATIP 866
MaHMGR  GMNMISKGTEKALLKLQEFF--PEMQILAVSGNYCTDKKPAAINWIEGRGKTVVCEAVIP 712
HsHMGR  GMNMISKGTEKALSKLHEYF--PEMQILAVSGNYCTDKKPAAINWIEGRGKSVVCEAVIP 713

                     1090      1100      1110      1120      1130      1140
        ....|....|....|....|....|....|....|....|....|....|....|....|
McHmgR1 SSVVQKVLKTTVEALVELNISKNLIGSAMAGSVGGFNAHAANILTAMYIAVGQDPAQNVE 962
McHmgR2 GAVVEKVLKTTVAALVELNVSKNLVGSAMAGSVGGFNAHAANILTAIYLATGQDPAQNVE 937
McHmgR3 GAVVEKVLKTTVAALVELNISKNLIGSAMAGSVGGFNAHAANILTAVYLATGQDPAQNVE 1001
RmHmgR  GPIVEKVLKTTVSALVELNTSKNLIGSAMAGALGGFNAHAANILTAIYLAAGQDPAQNVE 947
PbHmgR  GAIVEKVLKTTVAALVELNISKNLIGSAMAGSVGGFNAHAANILTAIYLATGQDPAQNVE 1054
SpHmgR  GDAVKSVLKTTVEDLVKLNVDKNLIGSAMAGSVGGFNAHAANIVTAVYLATGQDPAQNVE 928
ScHMG1  GDVVRKVLKSDVSALVELNIAKNLVGSAMAGSVGGFNAHAANLVTAVFLALGQDPAQNVE 930
ScHMG2  GDVVKSVLKSDVSALVELNISKNLVGSAMAGSVGGFNAHAANLVTALFLALGQDPAQNVE 926
```

```
MaHMGR   AKVVREVLKTTTEAMIDVNINKNLVGSAMAGSIGGYNAHAANIVTAIYIACGQDAAQNVG 772
HsHMGR   AKVVREVLKTTTEAMIEVNINKNLVGSAMAGSIGGYNAHAANIVTAIYIACGQDAAQNVG 773

                 1150      1160      1170      1180      1190      1200
         ....|....|....|....|....|....|....|....|....|....|....|....|
McHmgR1  SSNCITLMKSVN-NGSALHISCSMPS-IEVGTVGGGTILPPQQAMLDMLGVRGPHPTHPG 1020
McHmgR2  SSMCITLMKAVN-NDKDLNISCTMPC-IEVGTIGGGTILPPQQAMLDFLGVRGPHPTEPG 995
McHmgR3  SSNCITLMKAIN-NNQDLHISCSMPS-IEVGTIGGGTILPPQQAMLDMLGVRGPHPTEPG 1059
RmHmgR   SSNCITLMKAVN-DGKDLHISCTMPSHRKIGTVGGGTILPPQQAILDMLGVRGPHPTNPG 1006
PbHmgR   SSNCITLMKAVN-DTKDLHISCTMPS-IEVGTIGGGTILPPQQSMLDMLGVRGPHPTEPG 1112
SpHmgR   SSNCITLMDNVD---GNLQLSVSMPS-IEVGTIGGGTVLEPQGAMLDLLGVRGAHMTSPG 984
ScHMG1   SSNCITLMKEVD---GDLRISVSMPS-IEVGTIGGGTVLEPQGAMLDLLGVRGPHATAPG 986
ScHMG2   SSNCITLMKEVD---GDLRISVSMPS-IEVGTIGGGTVLEPQGAMLDLLGVRGPHPTEPG 982
MaHMGR   SSNCITLMEASGPTNEDLYISCTMPS-IEIGTVGGGTNLLPQQACLQMLGVQGACKDNPG 831
HsHMGR   SSNCITLMEASGPTNEDLYISCTMPS-IEIGTVGGGTNLLPQQACLQMLGVQGACKDNPG 832

                 1210      1220      1230      1240      1250      1260
         ....|....|....|....|....|....|....|....|....|....|....|....|
McHmgR1  KNAQKLARIICAAVMAGELSLCAALAAGHLVKAHMQHNRVSTAAAAAVPAVENDIHKKKA 1080
McHmgR2  ANARRLARIICASVMAGELSLCAALAAGHLVQAHMAHNRNTAAAAAAPAAAA----PSIA 1051
McHmgR3  KNAQRLARIICAAVMAGELSLCAALAAGHLVKAHMAHNR--------------------- 1098
RmHmgR   DNARRLARIICAAVMAGELSLCAALAAGHLVKAHMAHNR--------------------- 1045
PbHmgR   KNAQRLARIICAAVMAGELSLCAALAAGHLVKAHMAHNRGTQAPTIT------------- 1159
SpHmgR   DNSRQLARVVAAAVMAGELSLCSALASGHLVKSHIGLNRSALNTPAMDS----------- 1033
ScHMG1   TNARQLARIVACAVLAGELSLCAALAAGHLVQSHMTHNRKPA-EPTKPN----------- 1034
ScHMG2   ANARQLARIIACAVLAGELSLCSALAAGHLVQSHMTHNRK----TNKAN----------- 1027
MaHMGR   ENARQLARIVCGTVMAGELSLMAALAAGHLVRSHMVHNR--------------------- 870
HsHMGR   ENARQLARIVCGTVMAGELSLMAALAAGHLVKSHMIHNR--------------------- 871

                 1270      1280      1290
         ....|....|....|....|....|....|
McHmgR1  LKTPASTPRSLQSRQSTVPVTGNCIL---- 1106
McHmgR2  ASVIQASTPPATPVEKEDPIPGSCIKS--- 1078
McHmgR3  -----------AAAAATPAVPGTCIKS--- 1114
RmHmgR   --------------ATQAPAPGSCIKS--- 1058
PbHmgR   ----------SGPAPSTGTEPGTCIKS--- 1176
SpHmgR   ----------SAKKPATDALKSVNSRVPGR 1053
ScHMG1   ----------NLDATDINRLKDGSVTCIKS 1054
ScHMG2   ----------ELPQPSNKGPPCKTSALL-- 1045
MaHMGR   ------------SKINLQDLQGTCTKKSA- 887
HsHMGR   ------------SKINLQDLQGACTKKTA- 888
```

Figure S1 - Amino acid sequence of the three HMG-CoA reductases of *Mucor circinelloides* aligned to other known HmgR proteins. Red boxes show the sterol-sensing domains (SSD), green and blue boxes indicate the HMG-CoA-binding and the NAD(P)H-binding motifs, respectively. Grey and black filled characters represent the identical and the similar amino acids, respectively. Abbreviations and accession numbers of the amino acid sequences: McHmgR1, *M. circinelloides* HmgR1; McHmgR2 *M. circinelloides* HmgR2; McHmgR3 *M. circinelloides* HmgR3; RmHmgR, *Rhizomucor miehei* (CAE00496); PbHmgR, *Phycomyces blakesleeanus* (CAB97179); SpHmgR, *Schizosaccharomyces pombe* (AAB39277); ScHMG1 *Saccharomyces cerevisiae* (CAA86503); ScHMG2, *S. cerevisiae* (AAB67527); MaHMGR, *Mesocricetus auratus* (CAA25189); HsHMGR, *Homo sapiens* (NP_000850).

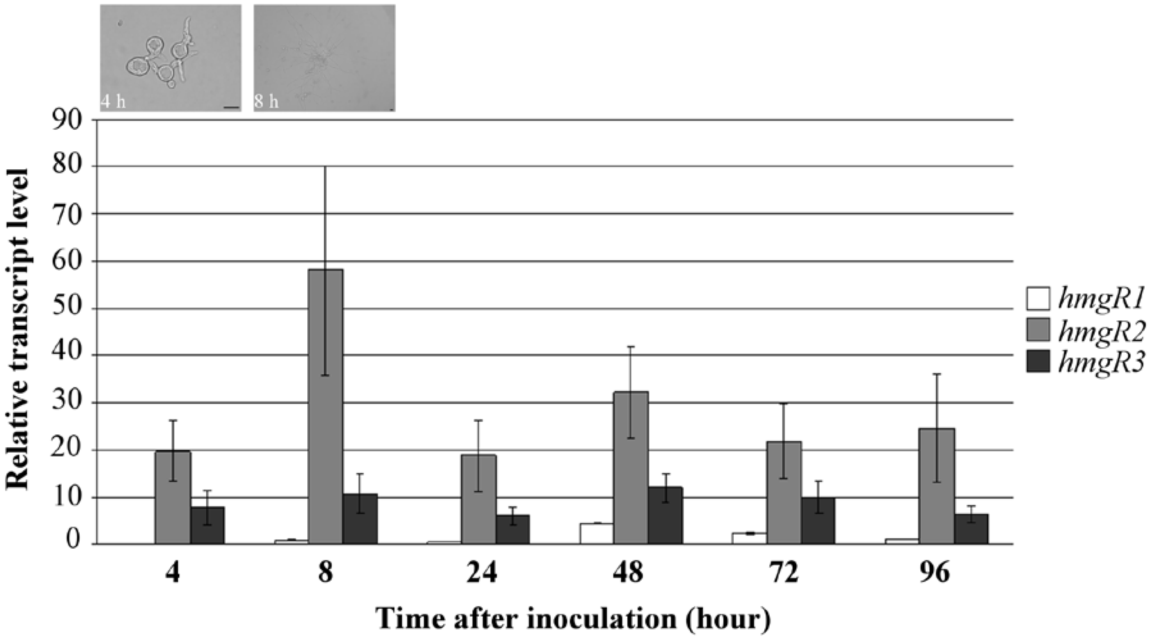

Figure S2 – Relative transcript levels of the *M. circinelloides hmgR* genes during the cultivation period. Relative transcript level of *hmgR1* at 96 hours was taken as 1. The presented values are averages of three independent experiments; the error bars indicate standard deviation. For RNA extraction, MS12 was cultivated on YNB under continuous light at 25 °C. Scale bars on the light micrographs indicate 10 µm.

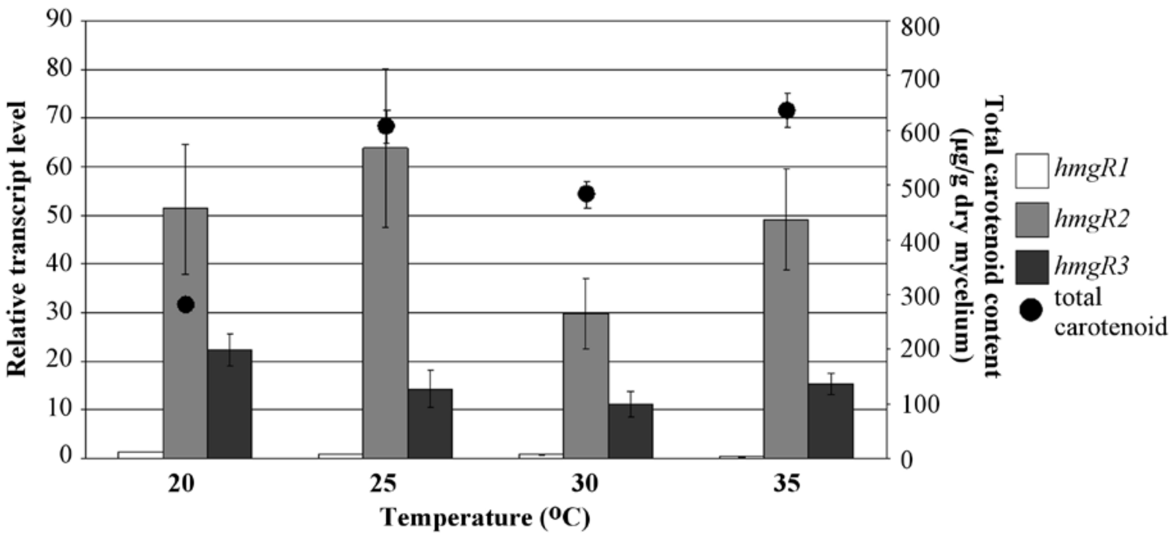

Figure S3 – Relative transcript levels of the *hmgR* genes and carotenoid content of *M. circinelloides* at different cultivation temperatures. Relative transcript level of *hmgR1* at 25 °C was taken as 1. The presented values are averages of three independent experiments; the error bars indicate standard deviation. For RNA and carotenoid extraction, MS12 was cultivated on YNB under continuous light for 4 days at different temperatures.

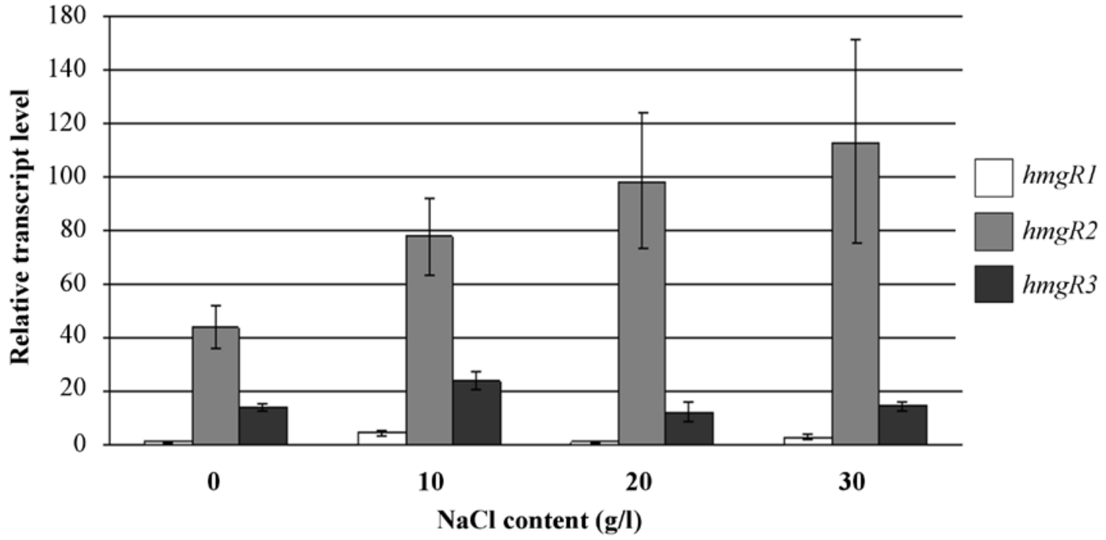

Figure S4 – Relative transcript levels of the *M. circinelloides hmgR* genes at different salt concentrations. Relative transcript level of *hmgR1* of the untreated control was taken as 1. The presented values are averages of three independent experiments; the error bars indicate standard deviation. For RNA extraction, MS12 was cultivated on YNB containing different concentrations of NaCl under continuous light for 4 days at 25 °C.

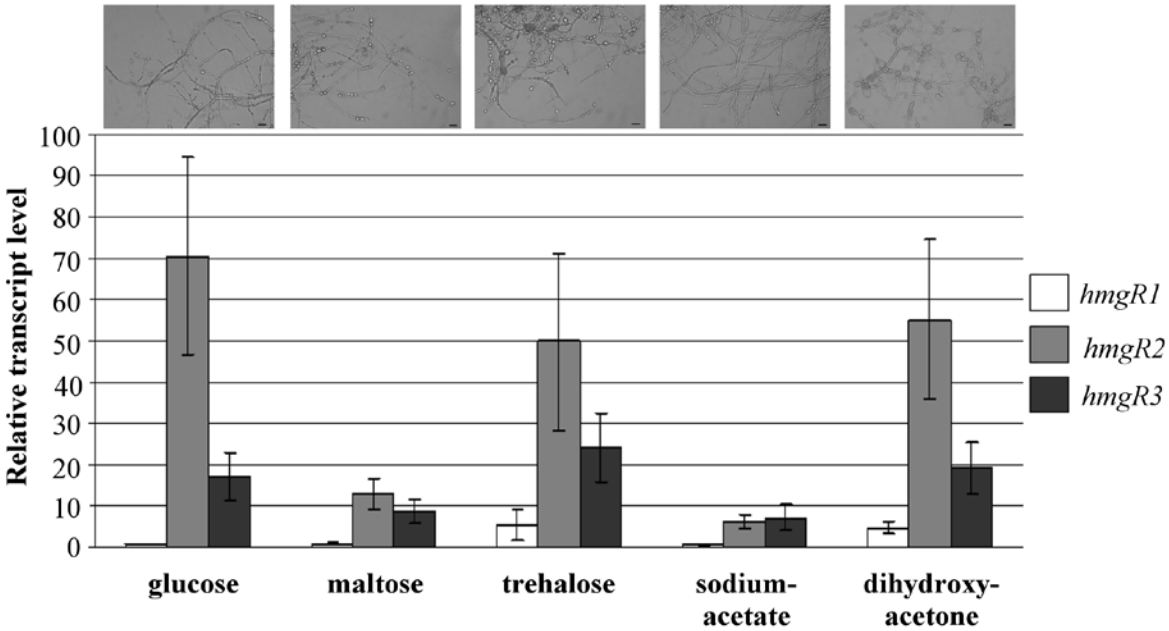

Figure S5 – Relative transcript levels of the *hmgR* genes of *M. circinelloides* growing on different carbon sources. Relative transcript level of *hmgR1* on YNB with glucose was taken as 1. The presented values are averages of three independent experiments; the error bars indicate standard deviation. For RNA extraction, MS12 was cultivated on YNB containing different carbon sources under continuous light for 4 days at 25 °C. Hyphal morphology on the different carbon sources are showed on the light micrographs; scale bars on the light micrographs indicate 10 μm.

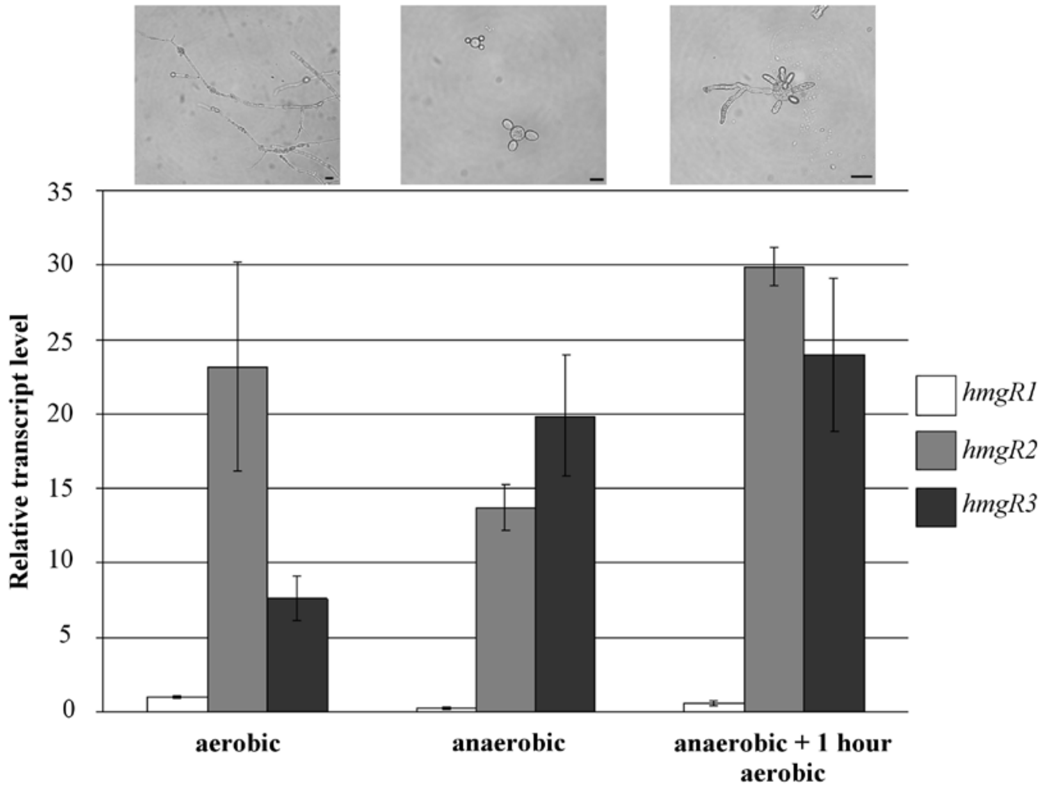

Figure S6 – Relative transcript levels of the *M. circinelloides hmgR* genes under aerobic and anaerobic growth conditions. Relative transcript level of *hmgR1* when the fungus was grown under aerobic condition was taken as 1. The presented values are averages of three independent measurements; the error bars indicate standard deviation. For RNA extraction, the MS12 strain was cultivated in liquid YNB under continuous light for 4 days at 25 °C. Morphology of MS12 under aerobic and anaerobic conditions are showed on the light micrographs, scale bars indicate 20 µm.

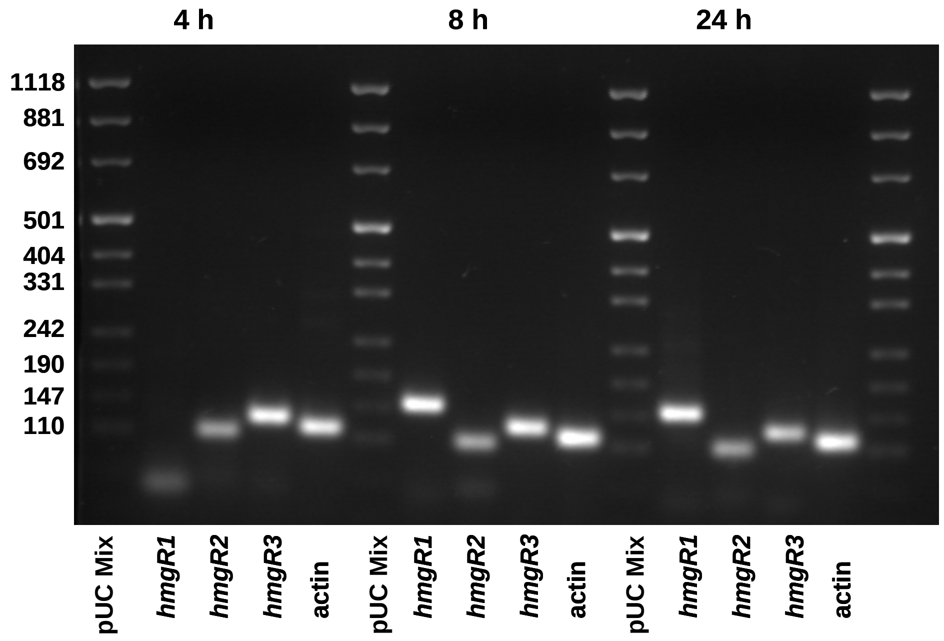

Figure S7 – Reverse transcription - PCR of the investigated genes. RNA extractions were performed after cultivations on YNB for the indicated times at 25 °C. PCR conditions and primers were the same as in the qPCR experiments. The pUC Mix (Fermentas) was used as a molecular weight marker. Size of the amplification products were as follows: *hmgR1*, 151 bp; *hmgR2*, 107 bp; *hmgR3*, 128 bp and *actin*, 117 bp.

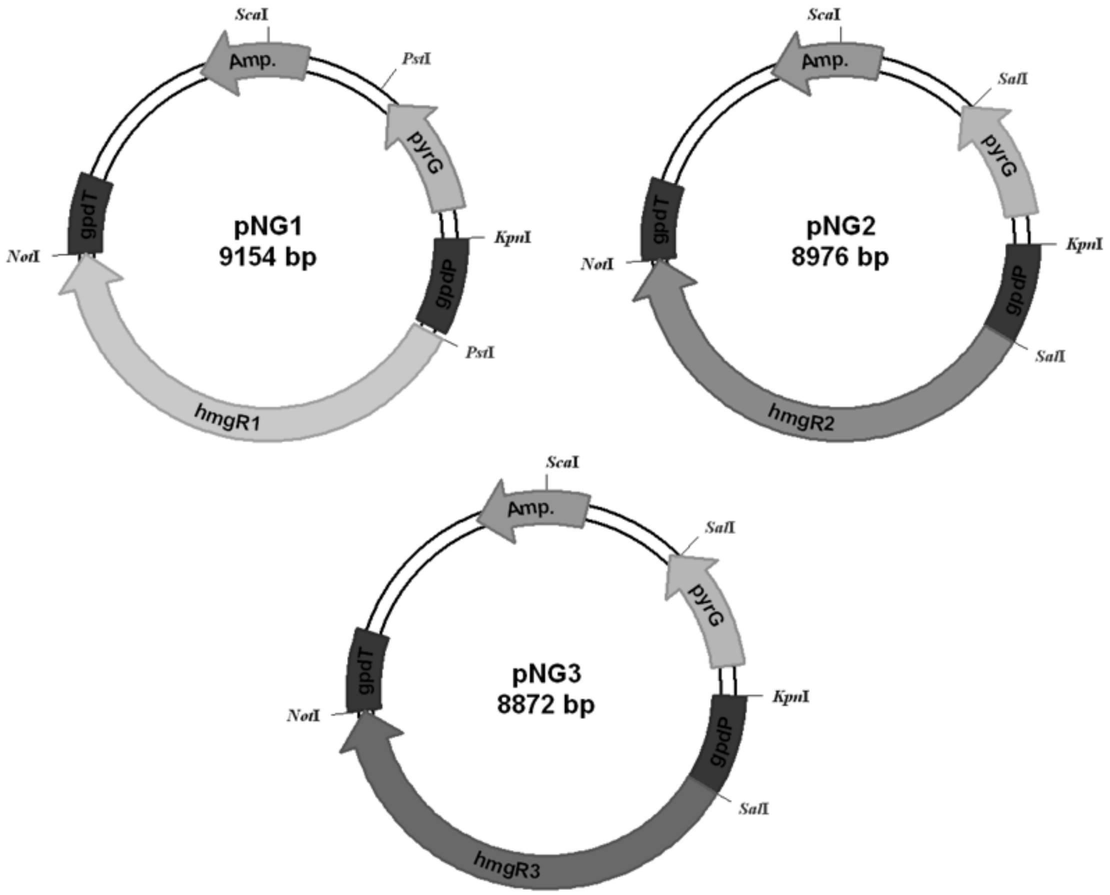

Figure S8 – Maps of the plasmids used in this study. Restriction sites used to construct the plasmids are shown.

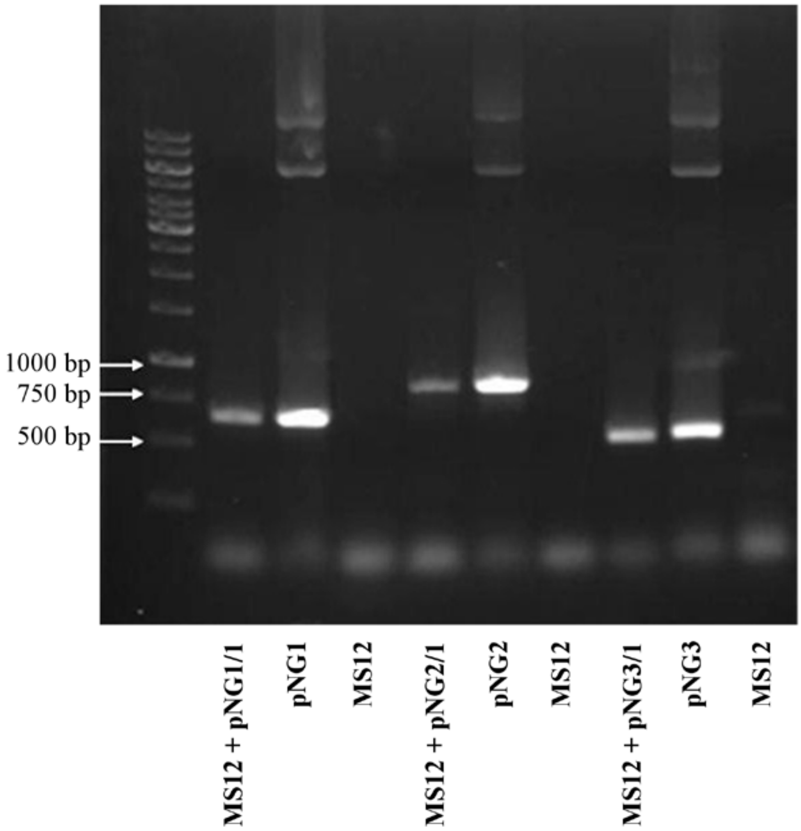

Figure S9 - PCR amplification of the transferred plasmids from the *M. circinelloides* transformants.

Primers used in these experiments were designed to the terminus of the *gpd*P (Gpdp) and the first part of each *hmgR* gene (png1rev for pNG1, png2rev for pNG2 and png3rev for pNG3). Sequences of the primers are shown in Table 1.

MS12+pNG1/1, MS12+pNG2/1 and MS12+pNG3/1, the transformants harbouring the indicated plasmids; pNG1, pNG2 and pNG3, the plasmids used in the study; MS12, the original, untransformed strain.
